# The role of lysine acylation in metabolic dysregulation and inflammatory responses within the hepatic immune microenvironment of MASLD

**DOI:** 10.3389/fimmu.2026.1933792

**Published:** 2026-09-03

**Authors:** Jingyi Peng, Ankangxin Yan, Guanglei Chen, Xiaojiao Gao, Qi Yu, Weiyi Tian, Kun Cai

**Affiliations:** 1 Guizhou University of Traditional Chinese Medicine, Guiyang, Guizhou, China; 2 Guizhou Nursing Vocational College, Qiannan Buyi and Miao Autonomous Prefecture, Guizhou, China; 3 Guiyang Kangyang University, Guiyang, Guizhou, China

**Keywords:** gut–liver axis, hepatic immune microenvironment, immunometabolism, inflammation, lysine acylation, MASLD

## Abstract

Metabolic dysfunction–associated steatotic liver disease (MASLD) is a chronic liver disorder characterized by metabolic abnormalities, persistent low-grade inflammation, and alterations in hepatic immune function. In addition to lipid accumulation and dysregulated energy metabolism, metabolism-associated post-translational modifications of proteins may also contribute to the initiation and progression of MASLD. Among these modifications, lysine acylation is regulated by the availability and composition of intracellular acyl-CoA species, as well as the activities of acyltransferases and deacylases, and can modulate metabolic enzyme activity, inflammatory signaling pathways, and gene transcription. This review summarizes the interplay between metabolic dysregulation and lysine acylation in MASLD, with particular emphasis on how gut-derived metabolites, disturbances in hepatic glycolipid metabolism, and alterations in subcellular metabolic environments influence the formation of distinct lysine acylation modifications. Furthermore, from the perspectives of hepatocytes, macrophages, hepatic stellate cells, and liver sinusoidal endothelial cells, we discuss the potential roles of lysine acylation in cellular injury, inflammatory responses, hepatic stellate cell activation, and liver fibrosis. In addition, we highlight the current progress regarding the potential of lysine acylation as a biomarker and therapeutic target, and propose that integrated approaches involving metabolic tracing, spatial omics, and site-specific genome editing may further elucidate the functional significance of key acylation events in MASLD. Overall, lysine acylation is closely associated with metabolic disturbances, hepatic inflammation, and fibrogenic processes in MASLD. A deeper understanding of cell type–specific regulatory acylation sites will facilitate the evaluation of their potential applications as biomarkers and therapeutic targets for MASLD.

## 1 Introduction

Metabolic dysfunction–associated steatotic liver disease (MASLD) is a chronic liver disorder primarily driven by metabolic abnormalities. With advances in disease understanding, MASLD is no longer considered merely a metabolic disorder; rather, it is increasingly recognized as a disease closely associated with persistent immune dysregulation and chronic inflammation (1). Epidemiological studies indicate that MASLD affects approximately 30% of adults worldwide and is frequently accompanied by obesity, type 2 diabetes mellitus, and cardiometabolic disorders (2–4). During disease progression, simple steatosis can further develop into metabolic dysfunction–associated steatohepatitis (MASH), liver fibrosis, and eventually hepatocellular carcinoma (HCC), thereby increasing the risk of end-stage liver disease and all-cause mortality (5, 6).

Accumulating evidence suggests that, in addition to increased metabolic stress, the initiation and progression of MASLD are closely associated with persistent activation of hepatic immune responses and functional alterations in immune cell populations. Under lipotoxic stress, hepatocytes can release damage-associated molecular patterns (DAMPs), which subsequently activate hepatic resident macrophages (Kupffer cells), infiltrating monocyte-derived macrophages, and T cells, thereby promoting the production of pro-inflammatory cytokines and chemokines (7). Consequently, sustained inflammatory responses further facilitate hepatic stellate cell (HSC) activation and their transition toward a myofibroblast phenotype, resulting in a multicellular pathological process characterized by sterile inflammation (8). Therefore, MASLD progression involves not only metabolic disturbances within the liver but also extensive alterations in immune cell activation, inflammatory signaling, and tissue injury.

Beyond nutritional excess and altered lipid metabolism, increasing evidence indicates that chronic neuroendocrine stress represents an additional upstream regulator of MASLD progression (9, 10). Persistent activation of the hypothalamic–pituitary–adrenal axis and sympathetic nervous system enhances hepatic substrate mobilization, mitochondrial metabolic burden, and oxidative stress, thereby reshaping intracellular metabolite availability. Excessive flux through glycolysis, fatty acid oxidation, and the tricarboxylic acid cycle increases the production of acyl-CoA metabolites, including acetyl-CoA, succinyl-CoA, malonyl-CoA, and lactate-derived lactyl-CoA, which serve as substrates for lysine acylation (11, 12). These stress-induced metabolic alterations may influence both hepatocytes and hepatic immune cells by modifying metabolic enzymes, transcriptional regulators, and inflammatory pathways. Moreover, neuroendocrine stress can promote systemic immune remodeling, including splenic myelopoiesis and monocyte recruitment, thereby establishing a feed-forward immunometabolic circuit that contributes to chronic hepatic inflammation (13, 14). Therefore, lysine acylation may represent an important molecular interface linking stress-induced metabolic flux with immune dysregulation during MASLD progression.

Recent advances in immunometabolism have demonstrated that metabolic alterations generate diverse intermediate metabolites that function not only as substrates for energy metabolism but also as signaling molecules regulating immune cell phenotypes and functions (15). Within the MASLD hepatic microenvironment, metabolic stressors, including nutrient overload, hypoxia, and lipotoxicity, induce metabolic remodeling in hepatocytes and multiple immune cell populations. These metabolic alterations are characterized by enhanced aerobic glycolysis accompanied by lactate accumulation; impaired mitochondrial function and disrupted tricarboxylic acid (TCA) cycle activity, leading to the accumulation of succinate and succinyl-coenzyme A (succinyl-CoA); increased *de novo* lipogenesis (DNL), resulting in elevated malonyl-CoA levels; and dysregulated ketogenesis, which alters β-hydroxybutyrate (β-HB) availability (16–18). Collectively, these metabolic changes reshape the composition and abundance of intracellular Acyl-coenzyme A (Acyl-CoA) pools across different subcellular compartments. Meanwhile, gut–liver axis-derived metabolites, including short-chain fatty acids (SCFAs) and bile acid derivatives, may further influence this metabolic regulatory network (12).

Alterations in the composition and abundance of intracellular acyl-CoA species provide metabolic substrates for the generation of various lysine acylation modifications, including lysine lactylation (Kla), lysine succinylation (Ksucc), lysine malonylation (Kmal), and β-hydroxybutyrylation (Kbhb). At the non-histone protein level, these acylation modifications can alter the electrostatic properties and conformational states of key lysine residues, thereby regulating the activity and molecular interactions of metabolic rate-limiting enzymes and immune-related signaling molecules, such as NF-κB and NLRP3 (12, 19, 20). At the epigenetic level, histone acylation can modulate chromatin accessibility and influence the transcription of genes involved in inflammation, metabolism, and fibrosis through the recruitment of acylation-recognizing proteins, including YEATS domain-containing proteins and members of the bromodomain and extraterminal (BET) family (21–25). More than ten types of lysine acylation modifications have been identified, including acetylation, succinylation, malonylation, lactylation, β-hydroxybutyrylation, crotonylation, and propionylation. However, the functional significance of these modifications varies considerably among disease contexts (12, 26). In MASLD, acetylation, succinylation, malonylation, lactylation, and β-hydroxybutyrylation have accumulated relatively stronger evidence linking metabolic alterations with hepatic inflammation and fibrosis (27, 28). Therefore, this review primarily focuses on these five major acylation types while discussing emerging modifications where relevant.

Metabolic reprogramming can regulate lysine acylation patterns and subsequently shape the functional states of immune cells. Therefore, this review discusses the impact of hepatic microenvironmental alterations on cellular functions and immune responses from the perspectives of metabolic dysregulation, lysine acylation, and immune regulation, and further explores the potential roles of lysine acylation in the initiation and progression of MASLD.

**Figure 1:**
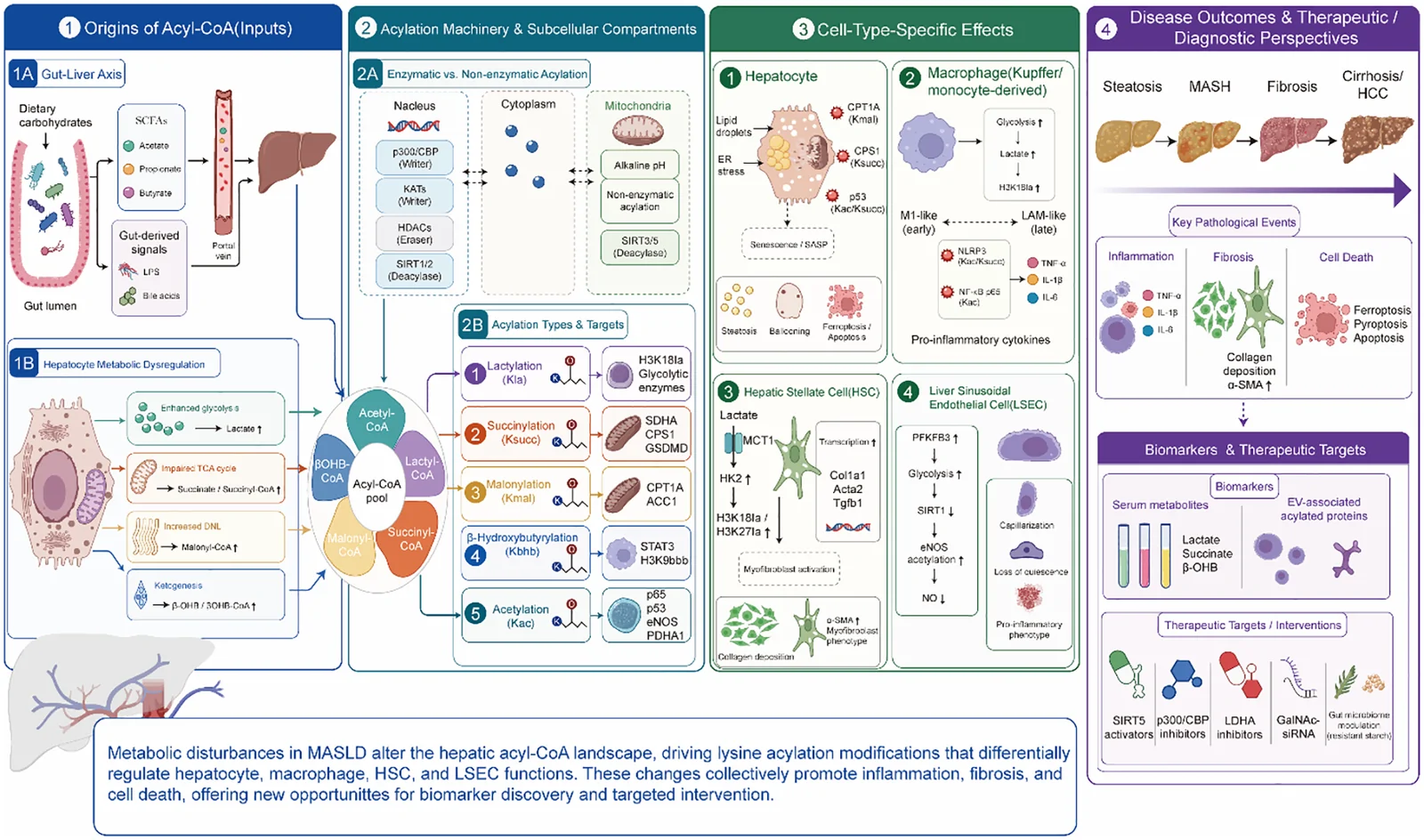
Metabolic regulation of lysine acylation in MASLD: origins, cell-specific functions, and implications for disease progression. This schematic summarizes the metabolic basis and biological consequences of lysine acylation remodeling during MASLD progression. Alterations in nutrient availability, mitochondrial dysfunction, and inflammatory stress modify intracellular acyl-CoA pools, including acetyl-CoA, succinyl-CoA, malonyl-CoA, lactyl-CoA, and β-hydroxybutyryl-CoA, thereby influencing different lysine acylation modifications. These modifications are regulated by acyltransferases (writers), deacylases (erasers), and acylation-binding proteins (readers), and subsequently affect metabolic enzymes, transcriptional regulators, and chromatin accessibility. In hepatocytes, macrophages, and hepatic stellate cells (HSCs), acylation remodeling contributes to lipid accumulation, inflammatory activation, immune-cell reprogramming, and fibrosis. The figure highlights the concept that lysine acylation functions as a metabolic sensor integrating intracellular metabolic status with hepatic immunometabolic responses during MASLD progression.

The development of high-throughput mass spectrometry technologies has provided powerful approaches for investigating acylation modifications associated with MASLD. To date, multiple acylation sites and alteration patterns have been identified in MASLD; however, most studies remain focused on the identification of modified sites and the characterization of global acylation landscapes. In contrast, the biological functions of specific acylation sites within distinct cell types and disease stages, as well as their causal relationships with MASLD development and progression, remain insufficiently understood. This knowledge gap has limited the translation of acylation-based discoveries into clinical applications. Therefore, this review systematically summarizes the roles of different lysine acylation modifications across various stages of MASLD progression and proposes a Level I–IV classification system for candidate acylation targets based on the strength of available functional evidence. Furthermore, by incorporating advances in liver-targeted delivery strategies, such as the asialoglycoprotein receptor (ASGPR)-mediated GalNAc delivery system, and emerging liquid biopsy-based biomarker approaches, we discuss the potential applications of lysine acylation in MASLD diagnosis and therapeutic intervention, providing insights for the development of future treatment strategies. An overview of the metabolic basis, cell-specific functions, and translational implications of lysine acylation in MASLD is shown in **Figure 1**.

## 2 Relationship between metabolic dysregulation and lysine acylation in MASLD

**Figure 2:**
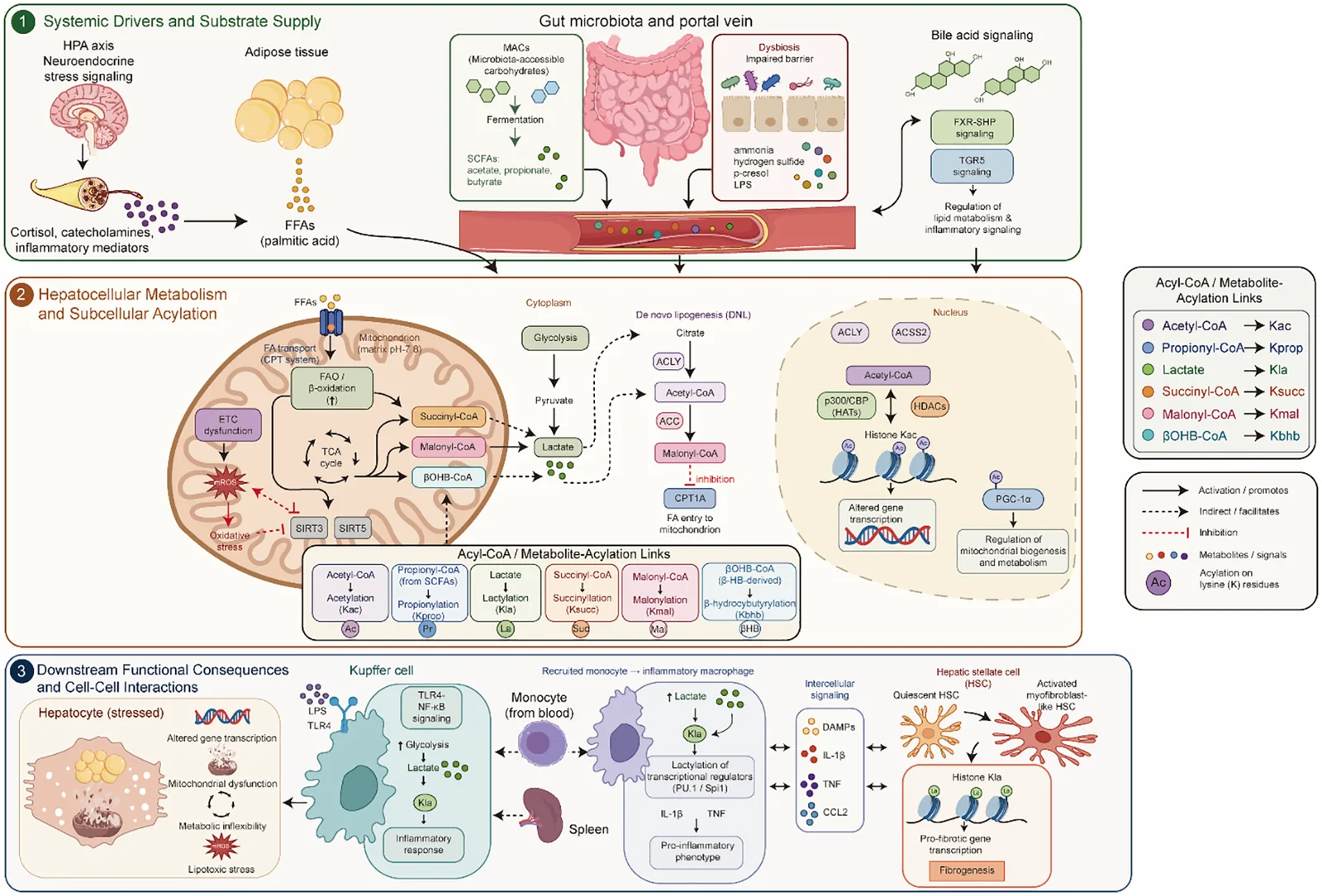
Metabolic reprogramming and lysine acylation-mediated regulation during MASLD progression. This schematic summarizes the relationship between systemic metabolic stress, acyl-CoA/metabolite availability, lysine acylation remodeling, and hepatic immunometabolic dysfunction during MASLD progression. Gut-derived metabolites, adipose-derived fatty acids, and neuroendocrine stress signals reshape hepatic metabolism and substrate flux, leading to alterations in acyl-CoA pools and corresponding lysine acylation events. These modifications regulate hepatocyte metabolism, mitochondrial function, inflammatory activation, and fibrotic responses through cell-specific mechanisms. The figure highlights acylation-mediated communication among hepatocytes, Kupffer cells, infiltrating macrophages, and hepatic stellate cells, ultimately contributing to steatosis, inflammation, immune activation, and fibrosis progression. Solid arrows indicate relatively established mechanisms, whereas dashed arrows represent proposed or incompletely validated pathways.

Alterations in lysine acylation are closely associated with the composition and abundance of intracellular acyl-CoA species. During the initiation and progression of MASLD, gut-derived metabolites entering the liver and metabolic disturbances in hepatic glycolipid metabolism can reshape the acyl-CoA pool within distinct subcellular compartments, thereby providing metabolic conditions for dysregulated lysine acylation. These acylation changes not only influence hepatocyte energy metabolism but may also regulate the activation states of hepatic immune cells and inflammatory responses. The integrated relationship between systemic metabolic stress, acyl-CoA availability, lysine acylation remodeling, and hepatic immunometabolic dysfunction is summarized in **Figure 2**.

### 2.1 Effects of gut-derived metabolites on hepatic acylation and immune responses

The gut microbiota can ferment microbiota-accessible carbohydrates (MACs) derived from dietary components and generate diverse metabolites. These metabolites can enter the liver through the portal circulation and provide exogenous substrates for hepatic acyl-CoA production. During MASLD development and progression, alterations in gut microbial composition and fermentation patterns lead to changes in the types and concentrations of metabolites delivered to the liver, thereby influencing hepatic metabolism and immune responses.

Under conditions of sufficient MAC availability, short-chain fatty acids (SCFAs), including acetate, propionate, and butyrate, represent major products of intestinal microbial fermentation. After entering the liver, acetate and propionate can be converted into acetyl-CoA and propionyl-CoA, respectively, thereby providing substrates for lysine acetylation (Kac) and lysine propionylation (Kprop). In addition to serving as an energy substrate, butyrate can inhibit the activity of class I and class II histone deacetylases (HDACs), thereby modulating the levels of histone acetylation, lactylation, and crotonylation. Consequently, butyrate-mediated epigenetic regulation may influence the transcription of immune-related genes and contribute to the maintenance of anti-inflammatory cellular phenotypes (29, 30).

Furthermore, SCFAs can regulate macrophage metabolic states and T-cell differentiation, thereby participating in the modulation of hepatic immune tolerance and inflammatory responses. Therefore, gut-derived metabolites function not only as metabolic substrates but also as regulatory signals that influence both innate and adaptive immune responses through effects on immune cell function.

When MAC availability is reduced or gut microbial dysbiosis occurs, intestinal fermentation may shift from carbohydrate-based fermentation toward protein fermentation, resulting in the production of metabolites such as ammonia, hydrogen sulfide, p-cresol, and lipopolysaccharide (LPS). Following disruption of the intestinal barrier, these metabolites, together with gut microbiota-derived extracellular vesicles (GMEVs), may more readily enter the liver through the portal circulation and induce mitochondrial stress and immune cell activation. Persistent ammonia overload and oxidative stress may impair the deacylase activity of SIRT3 and SIRT5, thereby promoting abnormal mitochondrial protein acylation. Meanwhile, LPS activates Kupffer cells through the TLR4–NF-κB signaling pathway, enhancing glycolysis and lactate production, which provides metabolic substrates for lysine lactylation (Kla) and promotes inflammatory responses (31, 32).

In addition to SCFAs, bile acids also participate in the regulation of hepatic metabolism and immune responses through the gut–liver axis. Alterations in intestinal pH and microbial composition can affect the abundance of bacteria capable of 7α-dehydroxylation, thereby modifying the ratio between primary and secondary bile acids (33, 34). The FXR–SHP and TGR5 signaling pathways regulate lipid metabolism and inflammatory responses and may indirectly influence hepatic protein acylation by modulating the expression or activity of acylation-related regulators, including the Sirtuin family and p300/CBP acetyltransferases.

### 2.2 Hepatocellular metabolic dysregulation and acyl-CoA accumulation

In addition to gut-derived metabolites, alterations in intrinsic hepatic glucose and lipid metabolism can also influence the composition and abundance of intracellular acyl-CoA species. During the development and progression of MASLD, lipotoxicity, mitochondrial dysfunction, and disturbances in glycolipid metabolism contribute to the accumulation of multiple acyl-CoA metabolites within hepatocytes, which may subsequently promote the formation of aberrant lysine acylation modifications.

Excessive free fatty acid (FFA) uptake, particularly palmitic acid, induces lipotoxic stress in hepatocytes and enhances fatty acid β-oxidation (FAO). Persistent activation of FAO increases the metabolic burden on the electron transport chain (ETC), resulting in elevated electron leakage and enhanced production of mitochondrial reactive oxygen species (mROS), thereby impairing mitochondrial respiratory function (35–37).

Furthermore, ETC dysfunction can disrupt the normal progression of the tricarboxylic acid (TCA) cycle. Impaired activity of key enzymes, including succinate dehydrogenase and α-ketoglutarate dehydrogenase, may lead to the intracellular accumulation of succinate and succinyl-CoA. Given that the mitochondrial matrix exhibits a relatively alkaline environment, increased levels of succinyl-CoA may facilitate the non-enzymatic formation of lysine succinylation (Ksucc). Such mitochondrial protein succinylation may affect the function of metabolic enzymes and further aggravate abnormalities in energy metabolism (38, 39).

In addition, under conditions of excessive glucose and lipid availability, *de novo* lipogenesis (DNL) is enhanced. Increased activity of acetyl-CoA carboxylase (ACC) promotes the production of malonyl-CoA. On the one hand, elevated malonyl-CoA inhibits carnitine palmitoyltransferase 1A (CPT1A), thereby reducing fatty acid transport into mitochondria for β-oxidation. On the other hand, malonyl-CoA can serve as a substrate donor for lysine malonylation (Kmal) and may influence mitochondrial oxidative metabolism (40).

Therefore, abnormalities in FAO, the TCA cycle, and DNL collectively reshape the composition and abundance of acyl-CoA species within distinct intracellular compartments of hepatocytes and may further regulate protein acylation events associated with metabolic adaptation and immune responses.

### 2.3 Relationship between different acyl-CoA species and lysine acylation

Dysregulation of hepatic glucose, lipid, and ketone body metabolism can alter the composition and abundance of acyl-CoA species within distinct subcellular compartments, thereby providing corresponding metabolic substrates for the formation of different types of lysine acylation. Alterations in specific acyl-CoA pools may further influence both histone and non-histone protein acylation patterns and are associated with metabolic disturbances, inflammatory responses, and hepatic fibrogenic processes.

In the nucleus and cytoplasm, acetyl-CoA is primarily generated by acetyl-CoA synthetase short-chain family member 2 (ACSS2) and ATP-citrate lyase (ACLY). Through the catalytic activity of acyltransferases, including p300/CBP, acetyl-CoA serves as a substrate for histone acetylation and regulates the expression of genes involved in metabolism and inflammation. Under nutrient overload conditions in MASLD, increased acetyl-CoA availability not only promotes lipid synthesis but may also regulate the acetylation status of metabolic regulators, such as peroxisome proliferator-activated receptor gamma coactivator 1α (PGC-1α), thereby affecting mitochondrial biogenesis and the metabolic states of immune cells (41, 42).

Enhanced glycolytic activity increases intracellular lactate production, thereby providing metabolic substrates for lactylation-related modifications and influencing the levels of histone lysine lactylation (Kla). Emerging evidence suggests that histone lactylation may participate in the transcriptional regulation of genes associated with inflammation and fibrosis, thereby affecting the functional states of macrophages and hepatic stellate cells (HSCs) (43).

Within mitochondria, impaired TCA cycle activity results in the accumulation of succinyl-CoA, which may increase lysine succinylation (Ksucc) levels and subsequently affect the function of proteins involved in oxidative phosphorylation. Meanwhile, enhanced *de novo* lipogenesis (DNL) increases malonyl-CoA availability. Malonyl-CoA can inhibit fatty acid β-oxidation (FAO) by suppressing carnitine palmitoyltransferase 1A (CPT1A) activity and may also contribute to the formation of lysine malonylation (Kmal), thereby further influencing mitochondrial metabolic function (40, 44).

Furthermore, β-hydroxybutyrate (β-HB), generated during ketone body metabolism, can be converted into β-hydroxybutyryl-CoA (βOHB-CoA) and participate in the formation of histone β-hydroxybutyrylation (Kbhb). This modification may be involved in cellular stress responses and the regulation of antioxidant-related gene expression (28, 45). The metabolic alterations, donor molecules, and regulatory characteristics of the major lysine acylation types are summarized in **Table 1**.

**Table 1:** Metabolic alterations, donor molecules, and regulatory characteristics of different lysine acylation modifications in MASLD.

Lysine acylation type	Associated metabolic alterations	Donor molecule	Major subcellular localization	Regulatory enzymes	Major associated hepatic cell types
Lysine lactylation (Kla)	Enhanced aerobic glycolysis and lactate accumulation	Lactyl-CoA	Nucleus, primarily involving histone modification; cytoplasm, mainly involving non-histone protein modification	Acyltransferase: p300/CBP; deacylases: HDAC1–3 and SIRT2	Activated hepatic stellate cells (aHSCs); pro-inflammatory macrophages
Lysine succinylation (Ksucc)	Mitochondrial dysfunction and impaired tricarboxylic acid (TCA) cycle activity, leading to the accumulation of succinate and succinyl-CoA	Succinyl-CoA	Mitochondrial matrix, predominantly generated through non-enzymatic reactions; nucleus	Formation mechanism: mainly non-enzymatic; nuclear regulation may involve KAT2A; deacylases: *SIRT5* (mitochondria) and SIRT7 (nucleus)	Lipotoxicity-induced injured hepatocytes; resting macrophages
Lysine malonylation (Kmal)	Enhanced *de novo* lipogenesis (DNL) and increased malonyl-CoA availability	Malonyl-CoA	Mitochondria and cytoplasm	Formation mechanism: potentially mainly non-enzymatic; specific acyltransferases remain to be identified; deacylase: *SIRT5*	Hepatocytes under nutrient overload conditions
Lysine β-hydroxybutyrylation (Kbhb)	Altered ketone body levels induced by fasting, ketogenic diets, or dysregulated ketone metabolism	β-Hydroxybutyryl-CoA (βOHB-CoA)	Nucleus, primarily involving histone modification; cytoplasm	Acyltransferase: p300/CBP; deacylases: HDAC1–3 and SIRT3	Hepatocytes under fasting conditions or with dysregulated ketone metabolism
Lysine acetylation (Kac)	Increased acetyl-CoA production mediated by ACSS2 or ACLY under conditions of dysregulated glucose and lipid metabolism	Acetyl-CoA	Nucleus, cytoplasm, and mitochondria	Acyltransferases: p300/CBP and KAT family members; deacetylases: SIRT1, SIRT2, SIRT3, and HDACs	Steatotic hepatocytes; liver sinusoidal endothelial cells (LSECs); activated hepatic stellate cells

This table focuses on five major lysine acylation modifications with relatively stronger evidence in MASLD-related metabolic regulation and immune remodeling, including acetylation, succinylation, malonylation, lactylation, and β-hydroxybutyrylation. Other modifications, such as lysine crotonylation and propionylation, have been identified in metabolic and epigenetic regulation but remain insufficiently characterized in MASLD.

**Figure 3:**
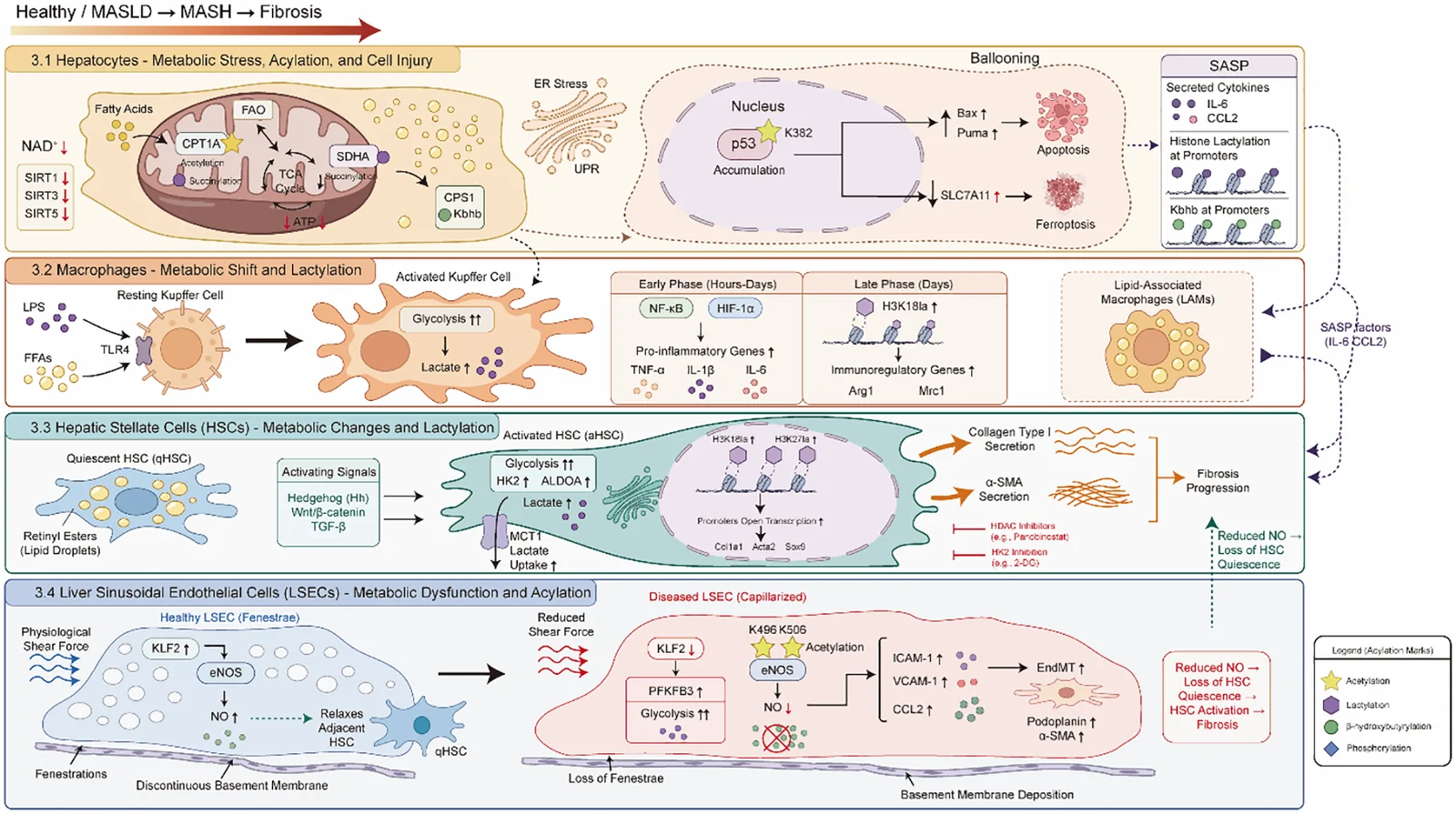
Alterations in lysine acylation patterns across different hepatic cell types in MASLD. This schematic depicts cell-specific functions of lysine acylation remodeling within the hepatic microenvironment. In hepatocytes, altered acyl-CoA metabolism affects mitochondrial enzymes, fatty acid oxidation, oxidative stress responses, and lipid accumulation. In Kupffer cells and infiltrating monocyte-derived macrophages, metabolic reprogramming and lactate accumulation promote histone and non-histone lactylation, inflammatory transcription, and cytokine production. In hepatic stellate cells, increased glycolysis-derived lactate contributes to histone lactylation and activation of profibrotic gene programs. Other immune populations, including neutrophils and lymphocyte subsets, may also undergo acylation-associated metabolic remodeling, although their specific roles in MASLD remain incompletely understood.

Therefore, alterations in different acyl-CoA species across distinct intracellular compartments can determine the availability of substrates for corresponding lysine acylation modifications and may contribute to the development and progression of metabolic abnormalities, inflammatory responses, and hepatic fibrosis in MASLD. Cell type-specific alterations in lysine acylation across the hepatic microenvironment are summarized in **Figure 3**.

### 2.4 Effects of subcellular compartments on lysine acylation

The occurrence and extent of lysine acylation are influenced by the characteristics of distinct subcellular compartments. The nucleus, cytoplasm, and mitochondria exhibit differences in pH, acyl-CoA availability, and the distribution of acyltransferases and deacylases. Therefore, the mechanisms underlying lysine acylation formation and the resulting modification patterns vary across different intracellular compartments.

Within the nucleus and cytoplasm, where the local environment is relatively neutral and free acyl-CoA concentrations are comparatively low, lysine acylation is predominantly regulated by enzymatic catalysis mediated by acyltransferases, including p300/CBP and members of the lysine acetyltransferase (KAT) family (46, 47). These enzymes recognize specific acyl-CoA substrates and catalyze the modification of corresponding lysine residues on target proteins (46, 48, 49). Meanwhile, metabolic enzymes such as ACSS2 and ACLY can contribute to local acetyl-CoA production within the nucleus, thereby maintaining nuclear acetyl-CoA availability and regulating histone acetylation and related gene transcriptional programs (50).

In contrast, the mitochondrial matrix exhibits a relatively alkaline environment, with a pH of approximately 7.8–8.0. Under these conditions, lysine residues are more prone to deprotonation, resulting in increased chemical reactivity (51). When reactive thioester metabolites, including succinyl-CoA and malonyl-CoA, accumulate within mitochondria, non-enzymatic lysine acylation may occur. If such modifications occur at critical sites of metabolic enzymes, they may affect mitochondrial respiratory chain activity and oxidative metabolic function (16, 17, 52–54).

In addition, mitochondrial deacylases, including SIRT3 and SIRT5, can remove specific acyl groups from mitochondrial proteins, thereby contributing to the maintenance of mitochondrial protein acylation homeostasis and metabolic function.

In MASLD, alterations in gut-derived metabolites, hepatic metabolic dysregulation, and acyl-CoA remodeling within different subcellular compartments may collectively influence the formation and distribution of lysine acylation modifications. Furthermore, dysregulated lysine acylation may subsequently affect hepatocyte metabolism, immune cell function, and inflammatory responses. Representative histone and non-histone lysine acylation events and their associated functional alterations are summarized in **Tables 2** and **3**.

**Table 2:** Histone lysine acylation and associated gene expression patterns across different stages of MASLD progression.

Pathological process	Major histone lysine acylation modifications	Associated genomic regions	Regulatory proteins	Representative genes	Disease stage and potential effects
Pro-inflammatory macrophage activation	H3K18la	Promoter regions of genes associated with inflammatory responses and macrophage polarization	p300/CBP; HDAC1–3	Tnf, Il1b, Il6	Inflammatory stage of MASH: may promote the expression of pro-inflammatory genes during the early inflammatory phase; with progressive lactate accumulation, may contribute to the expression of immune regulatory genes
Lipid-associated macrophage remodeling	H3K18la	Promoter regions of genes involved in immune regulation and tissue repair	Not yet defined	Trem2, Cd9, Arg1, Mrc1	Middle-to-late stages of MASH: may participate in macrophage transition toward lipid-associated macrophage (LAM) phenotypes and immune-regulatory states
Hepatic stellate cell activation and fibrosis	H3K18la, H3K27la	Promoter and enhancer regions of pro-fibrotic and extracellular matrix-related genes	YEATS domain-containing proteins, including AF9 and YEATS2; BRD4; P-TEFb	Col1a1, Acta2, Sox9, Tgfb1	Fibrotic stage: may increase chromatin accessibility at fibrosis-associated regulatory regions, thereby promoting hepatic stellate cell activation and pro-fibrotic gene expression
Hepatocyte senescence-associated secretory phenotype (SASP)	Kla, Kbhb	Promoter regions of genes associated with cellular senescence, inflammatory responses, and chemokine production	Not yet defined	Il6, Ccl2 (MCP-1)	Early MASLD stage: may contribute to the development of hepatocyte SASP and increase the expression of inflammatory cytokines and chemokines
Mitochondrial biogenesis and metabolic alterations	H3K122succ, H3K79succ	Genomic regions near transcriptional start sites of genes involved in energy metabolism and mitochondrial function	KAT2A, SIRT7, SIRT5	Genes associated with mitochondrial respiratory chain complexes	Different stages of MASLD progression: may influence nucleosome stability and the transcriptional regulation of mitochondria-associated genes

**Table 3:** Non-histone lysine acylation and associated protein functional alterations in MASLD.

Target protein	Modification site(s)	Lysine acylation type	Major subcellular localization	Regulatory enzymes or factors	Functional effects on proteins	Effects on proteins Potential roles in MASLD
CPT1A	K180, K291	Lysine malonylation (Kmal)	Outer mitochondrial membrane	SIRT5	CPT1A malonylation reduces its fatty acid transport activity	Reduced mitochondrial fatty acid uptake and β-oxidation leads to increased availability of fatty acids for triglyceride synthesis, thereby promoting lipid accumulation in hepatocytes
SDHA	Not defined	Lysine succinylation (Ksucc)	Mitochondrial matrix	SIRT5	SDHA succinylation decreases its catalytic activity	Impaired conversion of succinate to fumarate disrupts the tricarboxylic acid (TCA) cycle and promotes succinate accumulation. Increased extracellular succinate may further influence SUCNR1-mediated signaling
PDHA1	K321	Lysine acetylation (Kac)/lysine succinylation (Ksucc)	Mitochondrial matrix	p300, SIRT3	PDHA1 acylation affects its interaction with associated cofactors and reduces pyruvate dehydrogenase complex activity	Reduced conversion of pyruvate into acetyl-CoA decreases carbon flux into mitochondrial oxidative metabolism, while increased cytosolic pyruvate conversion to lactate may contribute to metabolic reprogramming
CPS1	Not defined	Lysine succinylation (Ksucc)	Mitochondrial matrix	SIRT5	Increased CPS1 succinylation decreases its catalytic activity	Impaired urea cycle activity reduces ammonia clearance, resulting in hepatic ammonia accumulation and potentially increasing mitochondrial reactive oxygen species production and mitochondrial permeability transition pore opening
RelA (p65)	K310	Lysine acetylation (Kac)	Nucleus	p300/CBP, SIRT1	p65 acetylation enhances its transcriptional activity and DNA-binding capacity	During MASH progression, reduced SIRT1 activity increases p65 acetylation, thereby promoting expression of inflammatory cytokines, including IL-6 and IL-1β
STAT3	K685	Lysine β-hydroxybutyrylation (Kbhb)/lysine acetylation (Kac)	Nucleus	p300, HDACs	Distinct acylation modifications at K685 regulate STAT3 homodimer formation and nuclear translocation	Ketogenic metabolic alterations may regulate inflammatory gene expression by modifying the acylation status of STAT3

### 2.5 Neuroendocrine stress-driven metabolic flux and acylation remodeling

Beyond local metabolic alterations within hepatocytes, increasing evidence suggests that systemic neuroendocrine stress may act as an upstream regulator of metabolic flux and lysine acylation remodeling during MASLD progression. Chronic activation of the sympathetic nervous system and hypothalamic–pituitary–adrenal (HPA) axis promotes glucocorticoid signaling and adipose tissue lipolysis, thereby increasing the delivery of free fatty acids and other metabolic substrates to the liver (9, 55). Consequently, excessive substrate influx challenges mitochondrial oxidative capacity and promotes the accumulation of acyl-CoA metabolites, including acetyl-CoA, succinyl-CoA, and malonyl-CoA, accompanied by increased reactive oxygen species (ROS) production (56).

These metabolic alterations provide a biochemical basis for widespread lysine acylation remodeling. Increased acetyl-CoA availability favors protein acetylation, whereas mitochondrial stress-associated accumulation of succinyl-CoA and malonyl-CoA may enhance lysine succinylation and malonylation of metabolic enzymes (11, 12, 57). In parallel, stress-induced glycolytic reprogramming increases lactate production, thereby promoting lysine lactylation of histone and non-histone proteins. Through these mechanisms, altered acylation patterns may influence key metabolic processes, including fatty acid oxidation, mitochondrial respiration, and inflammatory gene regulation (27, 58).

Importantly, the persistence of acylation remodeling may be reinforced by impaired mitochondrial deacylation capacity (59). Reduced activity of mitochondrial sirtuins, particularly SIRT3 and SIRT5, may limit the removal of acetyl, succinyl, and malonyl modifications from metabolic proteins (60). This imbalance between acylation and deacylation may contribute to sustained mitochondrial dysfunction, impaired metabolic flexibility, ROS accumulation, and increased susceptibility to lipotoxic stress in hepatocytes and hepatic immune cells.

In hepatic macrophages, chronic metabolic stress may induce a glycolytic phenotype characterized by increased lactate production and enhanced lactylation (61). Emerging studies indicate that lactylation can regulate macrophage transcriptional programs through modification of transcriptional regulators such as PU.1/Spi1, potentially influencing inflammatory activation and immune adaptation (62). Although direct evidence linking PU.1 lactylation to MASLD remains limited, this mechanism provides a potential molecular connection between metabolic reprogramming and persistent macrophage activation.

Furthermore, neuroendocrine stress-associated inflammatory remodeling may not be restricted to the liver. Hepatic release of DAMPs, together with inflammatory mediators such as IL-1β, TNF, and CCL2, may influence the liver–spleen axis by promoting splenic monocyte mobilization and inflammatory myelopoiesis (13). These recruited CCR2^+^ and CX3CR1^+^ monocytes subsequently migrate back to the liver, where they may further amplify Kupffer cell activation and hepatic stellate cell fibrogenic responses. Beyond serving as an immune-cell reservoir, the spleen represents an active immunometabolic organ that undergoes stress-induced remodeling (9, 63–65). Neuroendocrine signals and circulating inflammatory mediators can reshape splenic myelopoiesis and immune-cell activation states, involving metabolically primed monocyte subsets, myeloid-derived suppressor cells, and lymphoid populations such as natural killer T cells (14, 66). In addition, bioactive lipid mediators, including prostaglandin D2, leukotriene B4, sphingosine-1-phosphate, and endocannabinoid signals, may further regulate immune-cell trafficking and metabolic adaptation within the liver–spleen axis (67, 68). These systemic immune-metabolic alterations may interact with acyl-CoA remodeling and lysine acylation processes, thereby establishing a bidirectional regulatory circuit linking neuroendocrine stress, immune activation, and MASLD progression.

**Figure 4:**
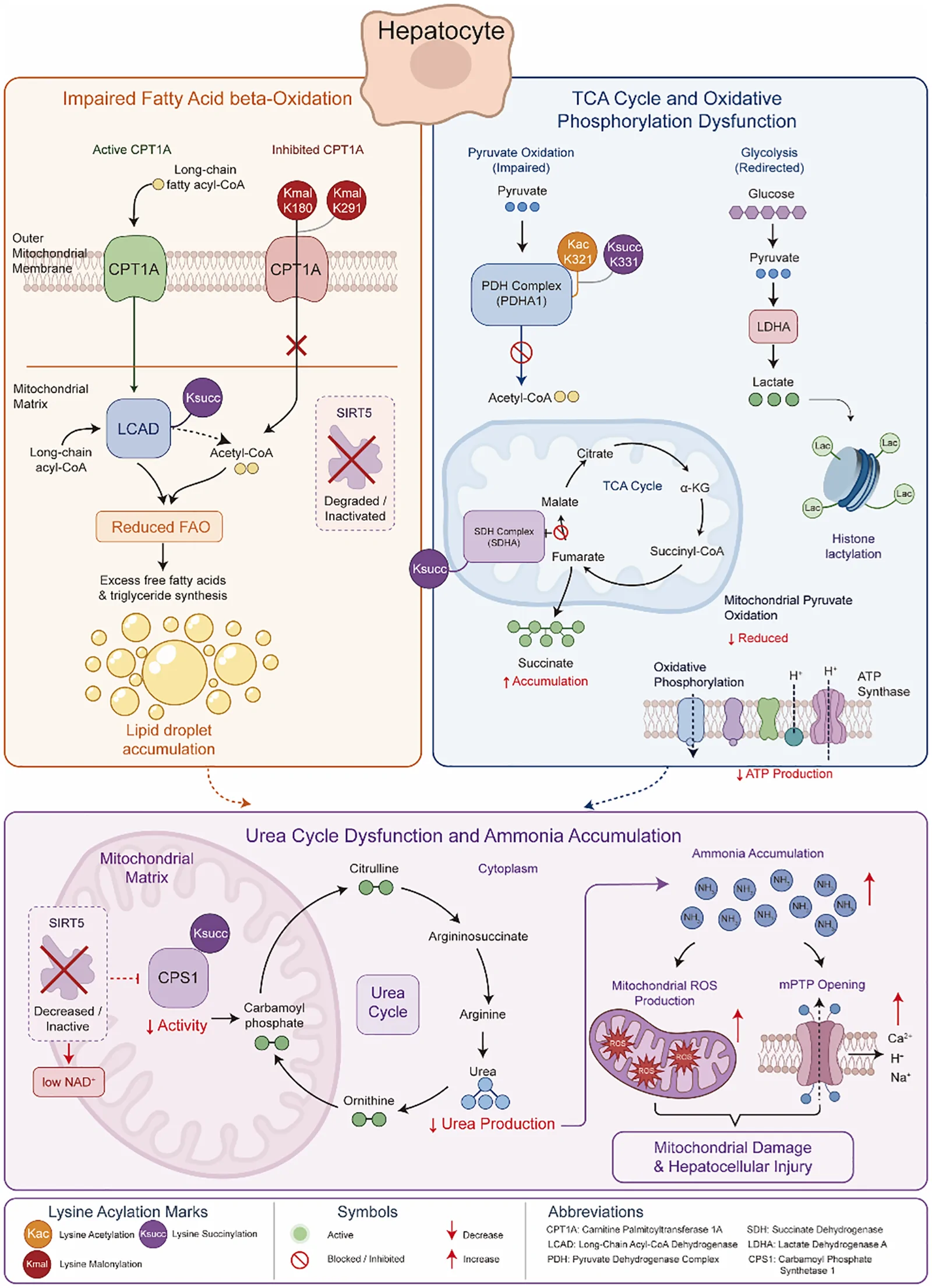
Lysine acylation-driven metabolic dysfunction during the early stage of MASLD. This figure summarizes how metabolic stress-induced lysine acylation contributes to early MASLD development. Excess nutrient influx promotes hepatic lipid synthesis, mitochondrial overload, and accumulation of acyl-CoA metabolites. Altered acetylation, succinylation, and malonylation of metabolic enzymes, including enzymes involved in fatty acid oxidation, the tricarboxylic acid cycle, and mitochondrial energy metabolism, impair metabolic flexibility and enhance lipid accumulation. Reduced activity of mitochondrial deacylases, particularly SIRT3 and SIRT5, further increases aberrant protein acylation, leading to mitochondrial dysfunction, oxidative stress, and hepatocyte metabolic injury.

In addition to hepatocytes, metabolic alterations associated with MASLD also reshape the functional state of hepatic immune and stromal cells. Metabolic intermediates derived from hepatocyte stress, including lactate, succinate, and other acyl-CoA-related metabolites, can influence macrophage activation, inflammatory signaling, and fibro-inflammatory responses through modulation of lysine acylation (27, 58). Similarly, immune populations such as infiltrating monocyte-derived macrophages, neutrophils, and lymphocyte subsets undergo metabolic adaptation during chronic liver inflammation, which may alter their acylation landscape and functional phenotypes. However, compared with hepatocytes and macrophages, the cell-specific functions of lysine acylation in other immune populations remain poorly characterized (12). These observations suggest that acylation remodeling represents an integrated immunometabolic process involving multiple hepatic cell populations rather than a hepatocyte-restricted event. The major acylation-associated metabolic abnormalities during the early stage of MASLD are summarized in **Figure 4**.

## 3 Alterations in lysine acylation across different hepatic cell types in MASLD

In MASLD, lysine acylation alterations occur in multiple hepatic cell populations. Cell type–specific changes in acylation patterns can influence hepatocyte metabolism and injury, macrophage activation and inflammatory responses, hepatic stellate cell (HSC) activation, and liver sinusoidal endothelial cell (LSEC) function, thereby potentially contributing to disease progression.

### 3.1 Metabolic stress, acylation alterations, and hepatocellular injury

Hepatocytes represent one of the major cell types exhibiting metabolic abnormalities during the early stages of MASLD. Dysregulated lysine acylation can affect the function of proteins involved in fatty acid oxidation, the urea cycle, and mitochondrial energy metabolism, including carnitine palmitoyltransferase 1A (CPT1A), succinate dehydrogenase subunit A (SDHA), and carbamoyl phosphate synthetase 1 (CPS1). Consequently, impaired activity of these metabolic regulators may reduce ATP production and contribute to hepatocellular metabolic dysfunction (69–72). Furthermore, the combined effects of energy metabolic disturbances and lipotoxic stress can induce endoplasmic reticulum stress (ER stress) and the unfolded protein response (UPR), which may contribute to hepatocellular ballooning degeneration during MASH development (73–76).

Under sustained metabolic stress, hepatocytes may also undergo senescence-associated alterations. In addition to reduced proliferative capacity, senescent hepatocytes acquire a senescence-associated secretory phenotype (SASP), characterized by the secretion of various inflammatory cytokines and chemokines (77–79). Previous studies have shown that histone lysine lactylation (Kla) and β-hydroxybutyrylation (Kbhb) levels can be increased at promoter regions of pro-inflammatory cytokine and chemokine genes, including Il6 and Ccl2, thereby altering chromatin accessibility and promoting the transcription and secretion of SASP-related factors (28, 58, 80). These secreted mediators can further act on neighboring Kupffer cells and hepatic stellate cells (HSCs), thereby promoting hepatic inflammation and fibrogenic responses.

In hepatocytes, deacylases including SIRT1, SIRT3, and SIRT5 contribute to the maintenance of protein acylation homeostasis in the nucleus, cytoplasm, and mitochondria (59, 81–83). During MASH progression, reduced NAD^+^ availability decreases SIRT1 activity, resulting in increased acetylation of p53 at lysine residues such as K382 (84). Enhanced p53 acetylation reduces Mdm2-mediated ubiquitination and degradation, thereby increasing p53 protein stability and promoting its nuclear accumulation. Nuclear accumulation of p53 subsequently induces the transcription of apoptosis-associated genes, including Bax and Puma, and may also promote ferroptotic hepatocyte death through the downregulation of SLC7A11.

### 3.2 Metabolic alterations and lactylation in macrophages

Kupffer cells and monocyte-derived macrophages both contribute to hepatic inflammatory responses during MASLD progression. Exposure to gut-derived lipopolysaccharide (LPS) and lipotoxic free fatty acids can promote the transition of macrophages toward a pro-inflammatory state, accompanied by increased production of inflammatory mediators, including tumor necrosis factor-α (TNF-α), interleukin-1β (IL-1β), and interleukin-6 (IL-6) (8, 85, 86). During this process, macrophage glycolytic activity is enhanced, leading to increased intracellular lactate accumulation.

Lactate accumulation promotes an increase in histone lysine lactylation (Kla), such as elevated histone H3 lysine 18 lactylation (H3K18la). During the early phase of inflammation, transcription factors including nuclear factor-κB (NF-κB) and hypoxia-inducible factor-1α (HIF-1α) primarily drive the expression of pro-inflammatory genes. However, with the persistence of inflammatory responses and further intracellular lactate accumulation, histone lactylation levels increase at the promoter regions of immune regulatory and tissue repair-associated genes, such as *Arg1* and *Mrc1 (*87–89). Enhanced histone lactylation can subsequently alter chromatin states at these regulatory regions and facilitate the transcription of genes involved in immune regulation and anti-inflammatory responses (87, 90–92).

**Figure 5:**
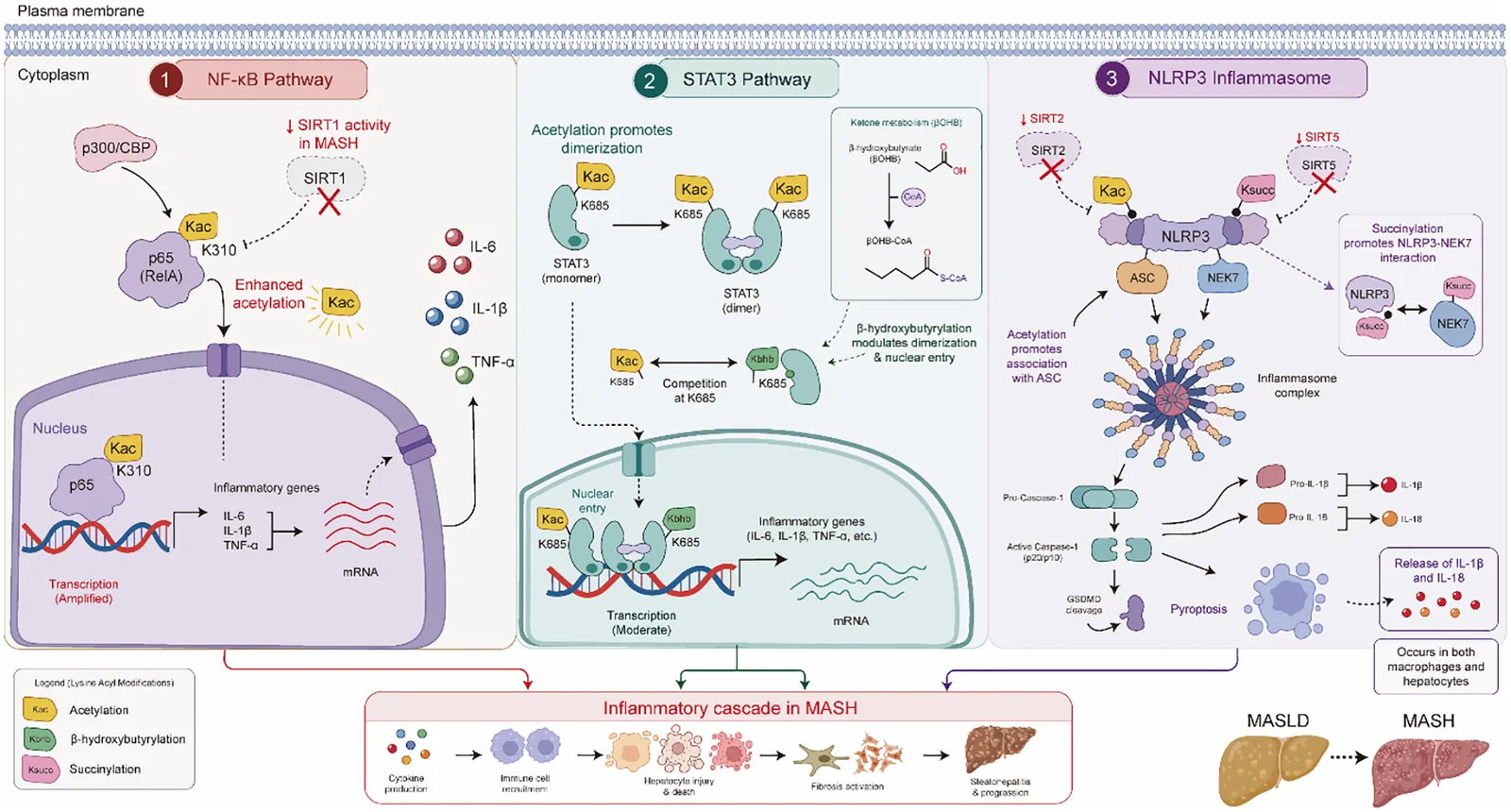
Lysine acylation-mediated regulation of inflammatory signaling during the progression from MASLD to MASH. This figure illustrates the contribution of lysine acylation to sterile inflammatory signaling during MASLD progression. Reduced deacetylase activity, including decreased SIRT1 and SIRT2 function, promotes acetylation of inflammatory regulators such as NF-κB p65 and NLRP3, enhancing inflammatory gene transcription and inflammasome activation. Metabolic stress-associated changes in β-hydroxybutyrate and other acyl-CoA metabolites may influence β-hydroxybutyrylation and acetylation of transcriptional regulators such as STAT3. These modifications promote production of pro-inflammatory mediators, including IL-1β, IL-6, IL-18, and TNF-α, thereby facilitating transition from metabolic dysfunction to chronic hepatic inflammation.

During sustained inflammatory activation and dynamic changes in gene expression profiles, a subset of macrophages may transition from a predominantly pro-inflammatory phenotype toward immune-regulatory or pro-fibrotic states, accompanied by the emergence of specialized macrophage populations, such as lipid-associated macrophages (LAMs) (27, 93, 94). Therefore, alterations in lactylation patterns may contribute to macrophage functional reprogramming during MASLD progression. Key lysine acylation-regulated inflammatory pathways during the progression from MASLD to MASH are summarized in **Figure 5**.

### 3.3 Metabolic alterations and lactylation in hepatic stellate cells

The transition of hepatic stellate cells (HSCs) from a quiescent state to an activated phenotype represents a critical pathological process underlying MASLD-associated liver fibrosis. Quiescent HSCs (qHSCs) contain retinyl ester-rich lipid droplets, whereas upon activation, they differentiate into myofibroblast-like cells (aHSCs) characterized by enhanced proliferative capacity, contractile activity, and extracellular matrix (ECM) production (70). During MASH progression, signaling pathways including Hedgehog, Wnt/β-catenin, and transforming growth factor-β (TGF-β) promote HSC activation. Concurrently, activated HSCs undergo metabolic reprogramming, characterized by enhanced glycolysis and glutaminolysis to meet the increased demands for cell proliferation, extracellular matrix synthesis, and energy production (95–97).

During HSC activation, increased glycolytic activity is accompanied by elevated intracellular lactate accumulation. Previous studies have demonstrated that activated HSCs exhibit increased expression of hexokinase 2 (HK2), which promotes glycolysis and lactate production and is associated with enhanced histone lactylation levels (27). In addition, the N6-methyladenosine (m6A) reader insulin-like growth factor 2 mRNA-binding protein 2 (IGF2BP2) can post-transcriptionally upregulate the expression of fructose-bisphosphate aldolase A (ALDOA), thereby further enhancing glycolytic flux and lactate generation (93, 98, 99). Increased lactate availability promotes the formation of lactyl-CoA and subsequently elevates histone lysine lactylation, particularly histone H3 lysine 18 lactylation (H3K18la) and H3 lysine 27 lactylation (H3K27la). Meanwhile, HSCs can uptake extracellular lactate through monocarboxylate transporter 1 (MCT1), thereby maintaining intracellular lactate homeostasis and modulating the expression of pro-fibrotic genes (95).

Studies have shown that H3K18la levels are increased at promoter and enhancer regions of fibrosis-associated genes, including *COL1A1*, *ACTA2*, and *SOX9*, accompanied by enhanced chromatin accessibility at these regulatory regions (100–102). This chromatin remodeling facilitates the recruitment of transcription factors and transcriptional complexes, thereby promoting the expression of pro-fibrotic proteins, including type I collagen and α-smooth muscle actin (α-SMA) (103). Genetic deletion of *Hk2* or inhibition of lactate production reduces histone lactylation levels and attenuates HSC activation and hepatic fibrosis. Furthermore, class I histone deacetylase (HDAC) inhibitors can increase H3K18 acetylation levels and may influence lactylation at the same lysine residue, thereby modulating the transcription of fibrosis-related genes (27).

**Figure 6:**
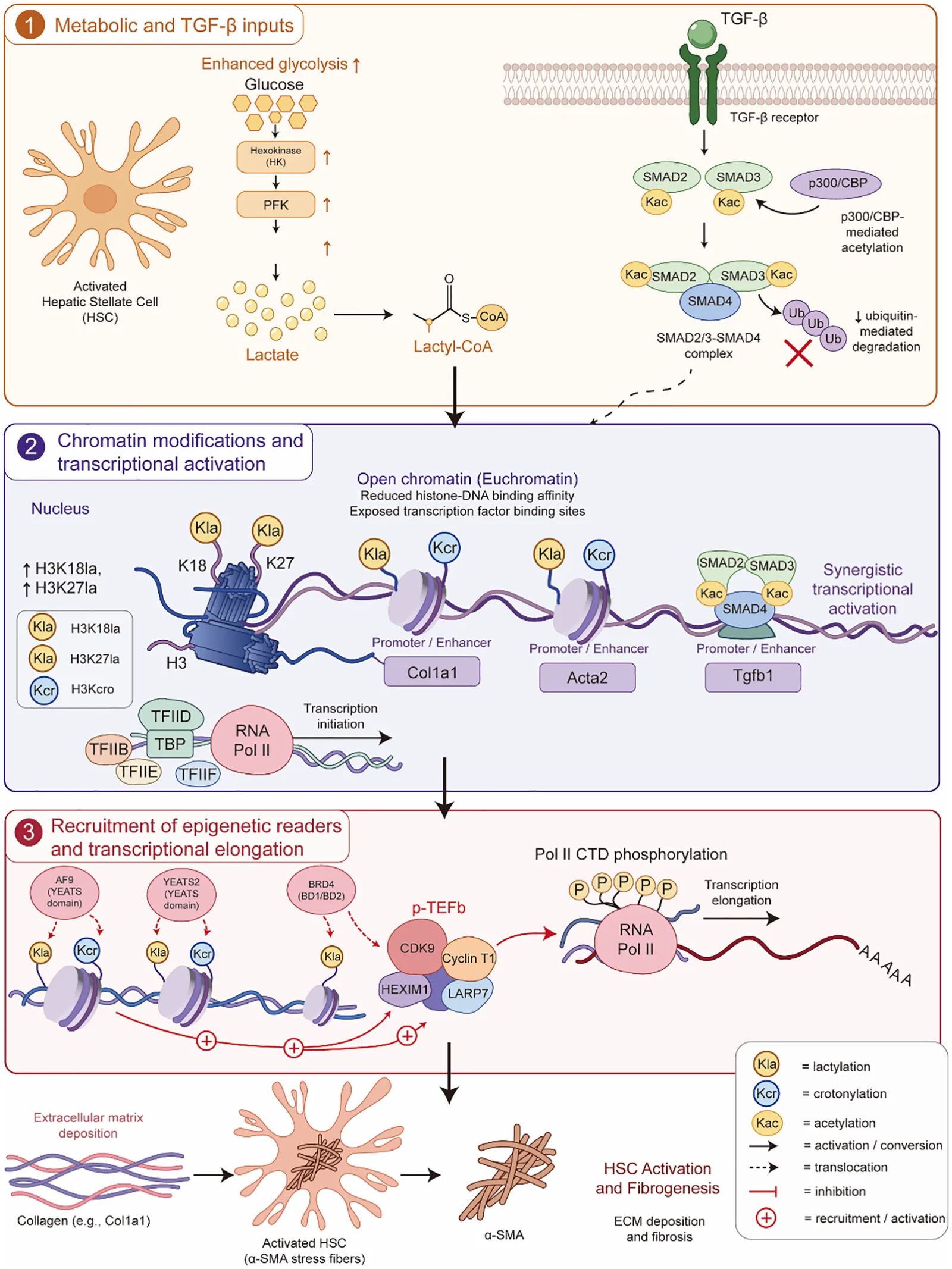
Lysine acylation remodeling and epigenetic readers promote hepatic stellate cell activation and fibrosis progression. This figure describes the role of lysine acylation in hepatic fibrosis development. Activation of hepatic stellate cells increases glycolysis and lactate production, resulting in elevated lactyl-CoA availability and enhanced histone lactylation, particularly H3K18la and H3K27la, at promoters or enhancers of profibrotic genes such as *Col1a1*, *Acta2*, and *Tgfb1*. Acylation-binding proteins, including YEATS domain-containing proteins and BRD4, recognize histone acylation marks and recruit transcriptional elongation machinery, thereby promoting extracellular matrix production and fibrosis-related transcriptional programs. Additional acetylation-dependent regulation of TGF-β/SMAD signaling may further enhance fibrogenic responses.

Therefore, *Hk2*, MCT1, and H3K18la may participate in HSC activation and hepatic fibrogenesis and represent potential candidate targets for therapeutic intervention in MASLD-associated liver fibrosis. Lysine acylation remodeling associated with HSC activation and fibrogenesis is summarized in **Figure 6**.

### 3.4 Metabolic dysregulation and acylation alterations in liver sinusoidal endothelial cells

Liver sinusoidal endothelial cells (LSECs) play essential roles in maintaining hepatic immune tolerance and microvascular homeostasis (104). Under physiological conditions, LSECs sense shear stress generated by portal blood flow, which promotes the expression of Krüppel-like factor 2 (KLF2), maintains endothelial nitric oxide synthase (eNOS) activity, and supports nitric oxide (NO) production. NO released from LSECs acts on neighboring hepatic stellate cells (HSCs) and contributes to the maintenance of their quiescent phenotype (105). During MASLD progression, intrahepatic lipid accumulation and microcirculatory disturbances alter sinusoidal shear stress, resulting in reduced levels of KLF2, eNOS, and NO (106). Consequently, LSECs undergo structural and functional alterations, including reduced or lost fenestrations, deposition of basement membrane components, and progressive capillarization (107).

Reduced KLF2 expression increases the expression of 6-phosphofructo-2-kinase/fructose-2,6-bisphosphatase 3 (PFKFB3), thereby promoting enhanced glycolytic activity in LSECs (108). In endothelial cells, the deacetylase SIRT1 mediates deacetylation of specific lysine residues on eNOS, including K496 and K506, facilitating the interaction between eNOS and calmodulin and maintaining NO production (109). Under MASH conditions, nutrient overload and oxidative stress reduce SIRT1 expression and activity in LSECs. Meanwhile, enhanced glycolytic activity may also be associated with increased eNOS acetylation levels (110, 111).

Increased eNOS acetylation impairs its enzymatic activity, resulting in reduced NO synthesis and release (112). As NO availability declines, the inhibitory effect of LSECs on HSC activation is attenuated, facilitating HSC transition toward a myofibroblast-like phenotype and increasing extracellular matrix deposition, thereby promoting hepatic fibrogenesis (105, 113). Furthermore, elevated PFKFB3 expression can enhance the expression of intercellular adhesion molecule-1 (ICAM-1), vascular cell adhesion molecule-1 (VCAM-1), and chemokine CCL2, while promoting endothelial-to-mesenchymal transition (EndMT). These changes contribute to enhanced pro-inflammatory responses and increased extracellular matrix production in LSECs (114).

Therefore, alterations in PFKFB3 expression, SIRT1 activity, and eNOS acetylation may collectively regulate LSEC metabolic states, NO bioavailability, and inflammatory responses, thereby contributing to the development of MASLD-associated hepatic fibrosis.

## 4 Lysine acylation and MASLD progression

During the development and progression of MASLD, lysine acylation not only represents a dynamic alteration in protein post-translational modification patterns but also regulates cellular metabolism, signal transduction, and gene transcription (47, 115). Therefore, lysine acylation may contribute to functional alterations across different cell types and subcellular compartments (116), and is associated with pathological processes including hepatic inflammatory responses, tissue remodeling, and cell death (12, 57, 117).

### 4.1 Lysine acylation alterations in energy metabolic dysfunction

During the early stages of MASLD, dysregulated lysine acylation can affect mitochondrial energy metabolism. Carnitine palmitoyltransferase 1A (CPT1A) is a key enzyme responsible for the transport of long-chain fatty acids into mitochondria for subsequent β-oxidation (118). Malonylation at specific lysine residues of CPT1A, including K180 and K291, can impair its transport activity (119, 120). Meanwhile, under conditions of impaired deacylase SIRT5 function, succinylation (Ksucc) levels of mitochondrial long-chain acyl-CoA dehydrogenase (LCAD) are increased, resulting in reduced enzymatic activity (119, 121). Dysfunction of CPT1A and LCAD collectively decreases fatty acid β-oxidation (FAO), thereby redirecting excessive free fatty acids toward triglyceride synthesis and promoting lipid accumulation in hepatocytes.

FAO impairment is frequently accompanied by disruption of the tricarboxylic acid (TCA) cycle and oxidative phosphorylation. Succinylation of succinate dehydrogenase (SDH), particularly its SDHA subunit, can affect the conversion of succinate to fumarate, thereby contributing to TCA cycle dysfunction and intracellular succinate accumulation (116, 122). The pyruvate dehydrogenase (PDH) complex serves as a critical metabolic link between glycolysis and the TCA cycle. Acetylation (Kac) or succinylation (Ksucc) at key lysine residues of its catalytic subunit PDHA1, including K321, can influence the interaction between PDHA1 and associated cofactors, thereby reducing the capacity of the PDH complex to catalyze the conversion of pyruvate into acetyl-CoA (123–125). When mitochondrial oxidative utilization of pyruvate is reduced, a proportion of pyruvate is redirected toward lactate production through lactate dehydrogenase A (LDHA), providing a metabolic substrate pool for histone lactylation and other lactate-derived modifications.

In addition to regulating fatty acid oxidation and the TCA cycle, dysregulated lysine acylation can also affect the urea cycle. Carbamoyl phosphate synthetase 1 (CPS1), a key enzyme in the urea cycle, catalyzes the conversion of free ammonia and carbon dioxide into carbamoyl phosphate. Under physiological conditions, SIRT5-mediated deacylation, particularly desuccinylation, contributes to the maintenance of CPS1 activity (126). During MASH progression, reduced NAD^+^ availability accompanied by decreased SIRT5 expression and activity leads to increased CPS1 succinylation and impaired enzymatic function. Consequently, reduced CPS1 activity compromises hepatic nitrogen waste disposal capacity, resulting in progressive ammonia accumulation within hepatocytes (127).

Elevated ammonia levels further promote mitochondrial reactive oxygen species (ROS) production and facilitate the opening of the mitochondrial permeability transition pore (mPTP), thereby exacerbating mitochondrial dysfunction (128).

### 4.2 Lysine acylation alterations in sterile inflammation-associated signaling pathways

During the progression from MASLD to MASH, lysine acylation can regulate hepatic inflammatory responses by modulating the activity of inflammation-associated signaling pathways, including NF-κB, STAT3, and NLRP3 inflammasome signaling.

The activity of the NF-κB pathway is closely associated with the deacetylase SIRT1. Acetylation of the NF-κB p65 subunit at lysine 310 (K310), mediated by p300/CBP, enhances its transcriptional activity and increases its binding capacity to target gene promoters. Under physiological conditions, SIRT1 mediates p65 K310 deacetylation in the nucleus, thereby suppressing the transcription of inflammatory genes (129). During MASH progression, reduced SIRT1 expression and activity lead to increased p65 acetylation, consequently enhancing the expression of inflammatory mediators, including IL-6, IL-1β, and TNF-α.

STAT3 dimerization and nuclear translocation are also regulated by lysine modifications. Acetylation of STAT3 at lysine 685 (K685) facilitates STAT3 homodimer formation and nuclear translocation (130). During MASLD progression, alterations in ketone body metabolism may affect intracellular β-hydroxybutyryl-CoA (βOHB-CoA) availability and potentially promote STAT3 β-hydroxybutyrylation (Kbhb). Because Kbhb and acetylation can occur at the same or adjacent lysine residues, changes in Kbhb levels may influence the acetylation status of STAT3, its homodimerization, nuclear localization, and subsequent regulation of inflammatory gene expression.

The assembly and activation of the NLRP3 inflammasome are similarly influenced by acetylation and succinylation modifications. NLRP3 acetylation promotes its interaction with apoptosis-associated speck-like protein containing a CARD (ASC) and facilitates inflammasome assembly, whereas SIRT2-mediated deacetylation suppresses this process (131–133). Under MASH conditions, reduced SIRT2 activity increases NLRP3 acetylation and promotes caspase-1 activation. In addition to acetylation, succinylation may also regulate NLRP3 inflammasome assembly and activity (134). SIRT5-mediated desuccinylation regulates the functions of NLRP3 and NEK7 and reduces the interaction between NEK7 and NLRP3 (135). Therefore, decreased SIRT5 expression or activity may increase the acylation levels of NLRP3 and NEK7, thereby promoting inflammasome activation, enhancing IL-1β and IL-18 release, and inducing pyroptotic responses in macrophages and hepatocytes.

### 4.3 Transcriptional regulation of pro-fibrotic genes

During hepatic fibrosis progression, hepatic stellate cells (HSCs) undergo enhanced glycolytic activity, resulting in increased intracellular lactate accumulation and the generation of lactyl-CoA, which subsequently influences histone lactylation levels (27). Levels of H3K18la and H3K27la at promoter and enhancer regions of pro-fibrotic genes, including *Col1a1*, *Acta2* (encoding α-smooth muscle actin), and *Tgfb1*, are increased during this process (93). These modifications reduce the interaction between histones and DNA, thereby increasing chromatin accessibility at regulatory regions. Consequently, enhanced accessibility facilitates the recruitment of transcription factors and RNA polymerase II, ultimately promoting the transcription of pro-fibrotic genes.

In addition to histone acylation, lysine acylation can also regulate SMAD2 and SMAD3, key mediators of transforming growth factor-β (TGF-β) signaling. p300/CBP-mediated acetylation of specific lysine residues on SMAD2/3 enhances their interaction with SMAD4 and reduces ubiquitin ligase-mediated degradation. As a result, SMAD2/3 protein stability and functional activity are increased, thereby promoting the expression of fibrosis-associated genes (136–138).

Histone acylation modifications can be recognized and bound by specific reader proteins. YEATS domain-containing proteins, including AF9 and YEATS2, recognize histone lysine lactylation (Kla) and crotonylation (Kcr) modifications and recruit positive transcription elongation factor b (P-TEFb), thereby facilitating RNA polymerase II transcriptional elongation (23). Bromodomain-containing protein 4 (BRD4) recognizes histone lysine acetylation (Kac) and promotes RNA polymerase II carboxy-terminal domain phosphorylation through recruitment of P-TEFb (139–141). Therefore, YEATS domain proteins and BRD4 participate in the transcriptional regulation of pro-fibrotic genes and are associated with HSC activation and increased extracellular matrix production.

### 4.4 Lysine acylation and hepatocyte death

During the late stages of MASLD progression, lysine acylation can regulate multiple forms of cell death, including ferroptosis, pyroptosis, and apoptosis, and is associated with hepatocellular injury.

Ferroptosis is a form of regulated cell death driven by excessive accumulation of lipid peroxides. Glutathione peroxidase 4 (GPX4) is a key enzyme responsible for eliminating lipid peroxides. Acetylation (Kac) or succinylation (Ksucc) of GPX4 has been reported to influence its protein stability in experimental models. However, whether these modifications directly promote GPX4 degradation through chaperone-mediated autophagy (CMA) in MASLD remains unclear. Given the critical role of GPX4 in limiting lipid peroxide accumulation, further studies are required to determine whether acylation-dependent regulation of GPX4 contributes to hepatocyte ferroptosis during MASH progression (142–145). Acyl-CoA synthetase long-chain family member 4 (ACSL4) promotes the incorporation of polyunsaturated fatty acids (PUFAs) into membrane phospholipids and contributes to ferroptosis susceptibility. In some experimental contexts, SIRT3 has been reported to regulate ACSL4 acetylation and activity (146). However, whether this regulatory mechanism operates in MASH hepatocytes remains to be experimentally validated. Although SIRT3 has been reported to regulate ACSL4 acetylation in other disease contexts, including cancer-related models, whether this regulatory mechanism operates in MASH hepatocytes remains to be experimentally validated. Therefore, ACSL4 acetylation represents a potential mechanism linking metabolic stress to ferroptosis susceptibility, but its specific contribution to MASLD progression requires further investigation (147, 148).

In addition to ferroptosis, excessive activation of the NLRP3 inflammasome can induce pyroptosis. Prior to activation by caspase-mediated cleavage, NLRP3 and the pyroptosis execution protein gasdermin D (GSDMD) can undergo lysine succinylation (Ksucc) or malonylation (Kmal). GSDMD succinylation has been proposed to influence its oligomerization and pore-forming activity in inflammatory models; however, whether this modification directly contributes to MASLD-associated pyroptosis remains insufficiently characterized (149). Reduced activity of deacylation enzymes, such as SIRT5 or SIRT2, may increase the acylation status of inflammatory proteins, potentially influencing inflammasome activation. However, the precise contribution of these modifications to MASLD progression remains to be clarified (131).

**Figure 7:**
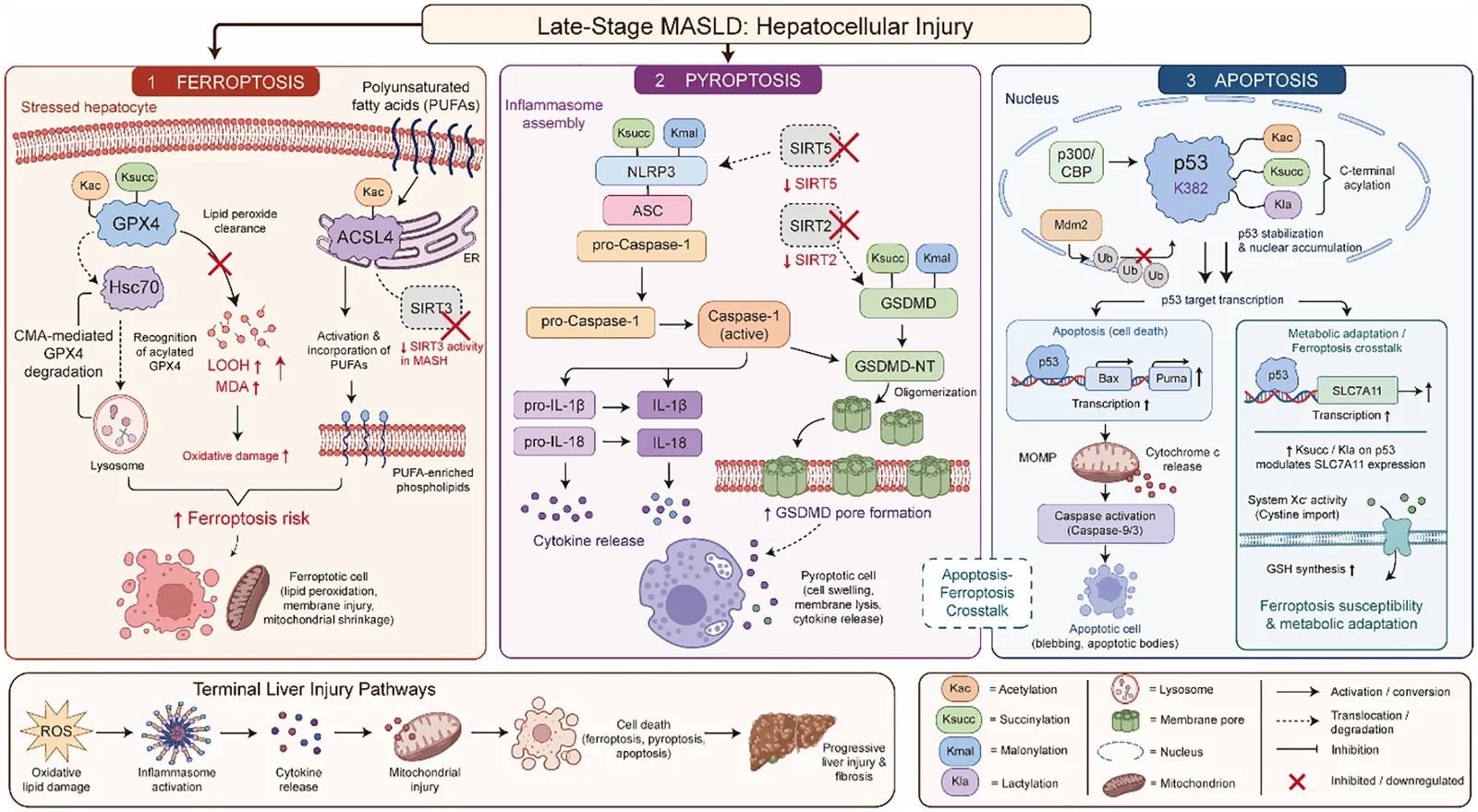
Lysine acylation-mediated regulation of ferroptosis, pyroptosis, and apoptosis during MASLD progression. This figure summarizes how lysine acylation influences hepatocyte death pathways in advanced MASLD. Altered acetylation and succinylation of antioxidant and metabolic regulators may impair lipid peroxide detoxification and increase susceptibility to ferroptosis. Changes in acylation status of inflammasome-related proteins, including NLRP3 and gasdermin D (GSDMD), may regulate inflammasome activation and pyroptotic cell death. In addition, acetylation, succinylation, and lactylation of apoptosis-associated regulators such as p53 may affect protein stability, transcriptional activity, and downstream expression of cell death-related genes. These mechanisms collectively contribute to hepatocyte injury, inflammatory amplification, and disease progression.

Furthermore, p53-mediated apoptosis is also regulated by lysine acylation. Acetylation of C-terminal lysine residues of p53, such as K382, mediated by p300/CBP, reduces Mdm2-dependent ubiquitination and degradation, resulting in increased p53 stability and nuclear accumulation. During MASLD progression, lysine residues of p53 may also undergo additional modifications, including succinylation (Ksucc) and lactylation (Kla), which together with acetylation regulate p53 stability, subcellular localization, and transcriptional activity (150). Different modification states may influence p53 binding to target gene promoter regions and regulate downstream gene expression. Increased p53 acetylation promotes the transcription of apoptosis-associated genes, including Bax and Puma. Meanwhile, elevated succinylation or lactylation may regulate genes such as SLC7A11, thereby influencing hepatocyte ferroptosis and metabolic adaptation (84). Lysine acylation-mediated regulation of ferroptosis, pyroptosis, and apoptosis during MASLD progression is summarized in **Figure 7**.

## 5 Intercellular communication in the liver and lysine acylation

During MASLD progression, lysine acylation not only participates in intracellular metabolic regulation and gene transcriptional control but may also be involved in intercellular communication mediated by metabolites, paracrine factors, and extracellular vesicles. Metabolic stress in hepatocytes can influence hepatic inflammation and fibrosis by modulating immune cell activation and hepatic stellate cell functions.

### 5.1 Paracrine interactions between hepatocytes and macrophages

In MASLD, injured hepatocytes can regulate the function of Kupffer cells and infiltrating macrophages through paracrine mechanisms. Following metabolic stress and steatosis-associated injury, disruption of the tricarboxylic acid (TCA) cycle in hepatocytes leads to intracellular succinate accumulation and its subsequent release into the extracellular environment. Extracellular succinate binds to succinate receptor 1 (SUCNR1, also known as GPR91) expressed on macrophages, activating Gq/11 protein signaling, calcium signaling, and MAPK pathways, thereby promoting the expression and secretion of pro-inflammatory cytokines by macrophages (151, 152).

Beyond paracrine metabolites, lipotoxic hepatocytes can also release extracellular vesicles (EVs). These EVs may contain integrins, lactate, succinate, and enzymes associated with glycolysis and mitochondrial metabolism (153, 154). Following uptake of hepatocyte-derived EVs by macrophages, the composition and abundance of intracellular acyl-CoA species may be altered, subsequently affecting the levels of lactyl-CoA and succinyl-CoA. These metabolic alterations may further regulate histone lysine lactylation (Kla) and non-histone lysine succinylation (Ksucc) in macrophages (155, 156).

Changes in macrophage acylation patterns may influence the expression of immunoregulatory genes and potentially contribute to macrophage transition toward lipid-associated macrophage (LAM) phenotypes. LAMs are typically characterized by high expression of TREM2 and CD9. Accumulation of LAMs in the liver is associated with local sterile inflammation, MASH progression, and hepatic fibrosis.

### 5.2 Lysine acylation-mediated immunometabolic communication within the hepatic microenvironment

During MASLD progression, lysine acylation should not be considered solely as a cell-autonomous regulatory mechanism, but rather as a molecular interface connecting metabolic stress with intercellular communication (12). Altered hepatic metabolism influences the production of extracellular metabolites, cytokines, DAMPs, and EVs, thereby reshaping the inflammatory microenvironment and coordinating responses among hepatocytes, immune cells, and stromal populations (157).

Hepatocyte-derived metabolic signals represent an important initiating event in this communication network (158). Lipotoxic stress, mitochondrial dysfunction, and altered substrate utilization modify intracellular acyl-CoA availability and reshape lysine acylation patterns within hepatocytes (159). Meanwhile, stressed hepatocytes release DAMPs, lactate, succinate, inflammatory mediators, and metabolite-enriched EVs, which can modulate immune-cell activation and propagate inflammatory responses. These signals may promote macrophage recruitment and metabolic adaptation, thereby establishing a feed-forward inflammatory circuit (8).

Kupffer cells and infiltrating monocyte-derived macrophages represent major immune responders to hepatocyte-derived metabolic stress (160). Exposure to extracellular metabolites, particularly lactate and succinate, may alter macrophage metabolic states and influence acylation-associated transcriptional programs. Increased glycolytic activity during inflammatory activation may enhance lactate availability and promote histone and non-histone lactylation, potentially sustaining inflammatory gene expression (58, 161). However, the specific contribution of individual acylation events to macrophage functional polarization in MASLD remains incompletely understood.

Beyond macrophages, multiple immune populations may participate in acylation-associated remodeling of the hepatic immune ecosystem. Neutrophils recruited during MASH progression undergo metabolic activation characterized by enhanced glycolysis, oxidative stress, and inflammatory mediator production, which may influence intracellular acyl-CoA metabolism and lactate-related acylation responses (62, 162). Similarly, CD4^+^ T cells, CD8^+^ T cells, and regulatory T cells undergo metabolic reprogramming during chronic liver inflammation, suggesting that lysine acylation may represent a potential mechanism linking nutrient availability with immune differentiation and effector functions. In addition, NK cells, NKT cells, dendritic cells, and B cells contribute to hepatic immune regulation through cytokine production and antigen presentation, although direct evidence connecting specific lysine acylation events with these populations remains limited (163).

Hepatic stellate cells integrate inflammatory and metabolic signals derived from surrounding cells and contribute to fibrosis progression. Crosstalk between hepatocytes, macrophages, and HSCs may create a reinforcing network in which inflammatory mediators and metabolic intermediates promote extracellular matrix deposition (27, 164, 165). Therefore, lysine acylation may function as a regulatory layer linking metabolic disturbances with multicellular communication during MASLD progression.

**Figure 8:**
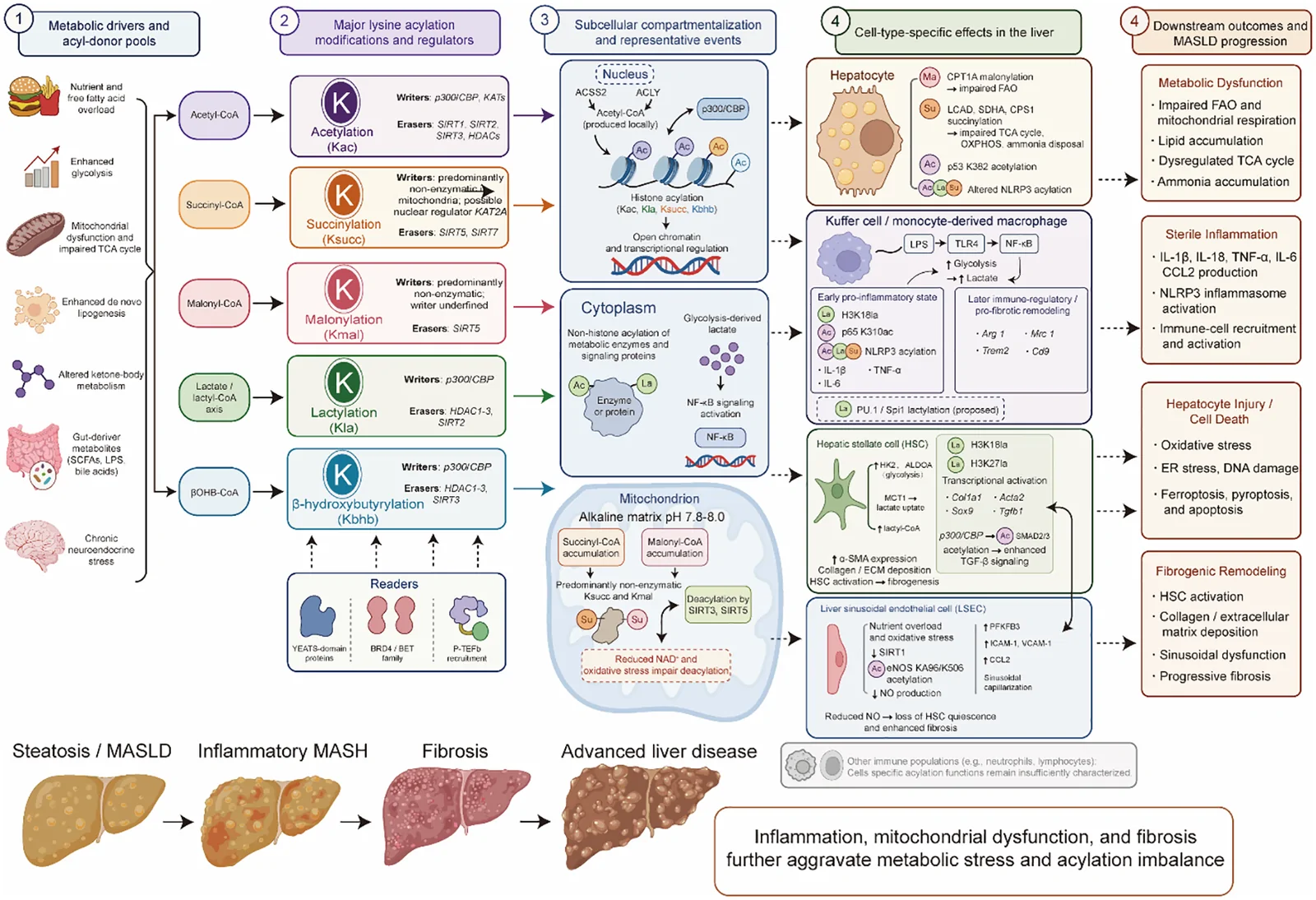
Lysine acylation links metabolic dysregulation to inflammation and fibrosis in MASLD. Metabolic stressors, including nutrient overload, mitochondrial dysfunction, altered glycolysis, gut-derived metabolites, and neuroendocrine stress, reshape intracellular acyl-CoA availability and drive diverse lysine acylation modifications. The figure summarizes five major acylation types with relatively established evidence in MASLD: acetylation (Kac), succinylation (Ksucc), malonylation (Kmal), lactylation (Kla), and β-hydroxybutyrylation (Kbhb), together with their representative writers, erasers, and regulatory mechanisms. Acylation remodeling occurs in distinct cellular compartments, including the nucleus, cytoplasm, and mitochondria, where it regulates chromatin accessibility, metabolic enzyme activity, inflammatory signaling, and mitochondrial homeostasis. Cell-specific effects are illustrated in hepatocytes, Kupffer cells/monocyte-derived macrophages, hepatic stellate cells, and liver sinusoidal endothelial cells, highlighting their contributions to metabolic dysfunction, sterile inflammation, hepatocyte injury, and fibrogenic remodeling. The overall framework depicts lysine acylation as a central immunometabolic regulatory network linking metabolic imbalance with inflammation and fibrosis during MASLD progression. Solid arrows indicate relatively well-supported mechanisms, dashed arrows indicate proposed or incompletely validated mechanisms.

Overall, acylation-mediated regulation represents a coordinated immunometabolic network rather than an isolated intracellular event. Future integration of single-cell acylome profiling, spatial multi-omics, and cell-type-specific genetic approaches will be required to determine how lysine acylation regulates hepatic intercellular communication across different stages of MASLD. An integrated framework linking metabolic dysregulation, lysine acylation, cell-specific responses, inflammation, and fibrosis in MASLD is shown in **Figure 8**.

## 6 Translational research and future perspectives

### 6.1 Approaches for validating causal relationships

Currently, research on MASLD-associated lysine acylation has largely focused on the identification of modification sites and the detection of acylation levels. To further determine whether alterations at specific acylation sites directly contribute to MASLD initiation and progression, future studies should integrate approaches including metabolic tracing, spatial omics, and genome editing.

First, stable isotope tracing can be applied to investigate the relationship between metabolic substrates and lysine acylation modifications. Researchers can utilize stable isotope-labeled substrates, such as [U-^13^C] -glucose and [U-^13^C] -lactate, in hepatic perfusion systems or animal models, followed by liquid chromatography–tandem mass spectrometry (LC–MS/MS) analysis to trace the incorporation of labeled carbon sources into metabolic pathways, acyl-CoA pools, and protein acylation events (58, 166). These approaches can determine whether specific metabolic substrates contribute to the formation of lysine acylation modifications and further define their metabolic origins.

Second, spatial omics and single-cell analyses provide powerful approaches for characterizing acylation alterations across distinct hepatic regions and cell populations. The liver exhibits pronounced metabolic zonation, with substantial differences in oxygen availability, blood flow, and metabolic activity between regions. Spatial proteomics and spatial analyses of protein post-translational modifications can preserve tissue architecture while enabling visualization of protein acylation patterns from the periportal region to the pericentral region. These approaches may further elucidate the relationships between oxygen gradients, hemodynamic factors, and cellular acylation states (167–169). In parallel, single-cell acylomics, or integrated analyses combining single-cell transcriptomics and proteomics, may facilitate the identification of cell type-specific acylation signatures in immune populations, including Kupffer cells and lipid-associated macrophages.

Finally, genome editing technologies can be employed to investigate the functional consequences of specific acylation sites. Conventional gene knockout or overexpression approaches may simultaneously alter multiple protein functions, making it difficult to distinguish the specific contribution of individual acylation sites. Depending on the target residue and experimental requirements, CRISPR/Cas9-based editing, base editing, or prime editing approaches can be used to generate site-specific lysine substitution models, such as lysine-to-arginine or lysine-to-alanine mutations. Subsequent evaluation in cellular and animal models can reveal the effects of individual modifications on metabolic dysfunction, inflammation, and fibrosis-related phenotypes (170). Although delivery systems such as lipid nanoparticles have shown potential for liver-targeted gene editing, their efficiency and cell-type specificity among different hepatic populations require further investigation.

Collectively, the integration of metabolic tracing, spatial analyses, and site-specific functional validation will help establish mechanistic links among metabolic alterations, lysine acylation remodeling, and cellular functional changes. These strategies will provide a foundation for identifying clinically relevant acylation targets and developing therapeutic interventions for MASLD.

### 6.2 Evidence-based classification of lysine acylation targets

With the rapid development of proteomic and post-translational modification profiling technologies, numerous lysine acylation sites have been identified in MASLD. However, the functional significance and translational potential of individual modification sites vary considerably, and many studies remain limited to associations between acylation alterations and disease phenotypes. To facilitate comparison of the current evidence supporting different acylation targets, this review classifies candidate targets into four levels based on functional validation, clinical relevance, and site-specific *in vivo* mutation studies. This classification summarizes the strength of available evidence and provides a framework for prioritizing future target discovery and therapeutic evaluation (12, 116).

Level I: Targets supported by relatively comprehensive evidence. Targets in this category should demonstrate consistent acylation alterations in liver tissues from patients with MASH and be validated in at least two independent animal models. Furthermore, site-specific mutation experiments, such as lysine-to-arginine substitution models, should confirm whether modification of the specific residue is sufficient to improve or reverse disease-associated phenotypes. Acylation-mediated regulation of specific lysine residues in CPT1A represents a representative example of this category. Current evidence suggests that targeting these modification sites may ameliorate hepatic steatosis and metabolic dysfunction (57, 116).

Level II: Targets with substantial functional evidence. Targets in this category exhibit correlations between modification levels and disease severity in patient samples, such as associations with MASH inflammatory scores or fibrosis stages. Corresponding phenotypic improvements have also been observed in knockout or transgenic animal models. However, direct *in vivo* validation through endogenous site-specific mutation remains lacking. Studies involving NLRP3 acetylation or succinylation belong to this category, as their associations with inflammasome activation and hepatic inflammation have been extensively reported.

Level III: Candidate targets supported by cellular and animal studies. Targets in this category have demonstrated potential therapeutic effects in animal models through pharmacological intervention, siRNA-mediated suppression, or other non-site-specific approaches, with proposed mechanisms supported by cellular experiments. Nevertheless, direct evidence from human tissues remains limited, preventing definitive conclusions regarding their association with MASLD severity. Several studies investigating mitochondrial dehydrogenase succinylation and its relationship with TCA cycle dysfunction may be classified within this category (116).

Level IV: Targets at an early exploratory stage. Targets in this category are primarily investigated using *in vitro* models, including hepatocyte and macrophage cell lines, often under experimental conditions involving high glucose, high lipid exposure, or palmitate/oleate stimulation. Currently, validation in animal models and clinical samples remains insufficient. Studies examining the relationship between specific STAT3 lysine acetylation or β-hydroxybutyrylation sites and STAT3 dimerization and nuclear translocation largely remain within this category (130).

This evidence-based classification enables discrimination among lysine acylation targets according to their current levels of mechanistic and translational support. Level I and Level II targets, supported by relatively strong functional evidence and disease relevance, represent promising candidates for drug development and *in vivo* intervention studies. In contrast, Level III and Level IV targets require further validation using patient samples, animal models, and site-specific functional approaches. Such a framework may facilitate the transition of MASLD-related lysine acylation research from descriptive modification profiling toward mechanistic validation and therapeutic target evaluation (171). The proposed evidence-grading framework and representative candidate targets are summarized in **Table 4**.

**Table 4:** Evidence grading and representative candidate targets of lysine acylation in MASLD.

Evidence level	Current research basis and translational potential	Major criteria for classification	Applicable validation approaches	Representative candidate targets or sites
Level I	Strong evidence with relatively high translational potential	1. Consistent alteration of modification levels observed in liver tissues from patients; 2. Validation obtained in at least two independent animal models; 3. Direct association between specific modification sites and disease phenotypes demonstrated by *in vivo* site-specific mutation experiments.	Lysine site-specific knock-in mouse models, such as K-to-R substitution models; liver-targeted lipid nanoparticle delivery of base editing systems; pathological evaluation of patient liver tissues and clinical cohort analyses.	Specific acylation or malonylation sites of CPT1A
Level II	Substantial functional evidence, but requiring further site-specific validation	1. Modification levels in patient tissues are correlated with disease severity; 2. Phenotypic improvement observed in global or conditional gene knockout animal models; 3. Lack of *in vivo* validation using endogenous site-specific mutation models.	Conditional gene knockout mouse models, such as Flox/Cre systems; evaluation of MASH inflammatory scores, liver fibrosis staging, and related pathological parameters.	Acetylation or succinylation modifications associated with the NLRP3 inflammasome
Level III	Primarily supported by animal and cellular experimental evidence	1. Partial phenotypic improvement observed through non-site-specific interventions in animal models; 2. Potential mechanisms proposed using primary cells or cellular models; 3. Lack of direct evidence from patient tissues or clinical cohorts.	Small-molecule intervention, siRNA-mediated knockdown, primary cell culture, metabolite profiling, and protein modification analysis.	Succinylation of mitochondrial dehydrogenases and related mechanisms involving tricarboxylic acid cycle dysfunction
Level IV	At an exploratory and preliminary research stage	1. Studies mainly limited to cell lines such as Hepa1–6 and RAW264.7; 2. *In vitro* models primarily established using high-glucose, high-fat, or palmitic acid/oleic acid stimulation; 3. Lack of validation in animal models and clinical samples.	Cell culture experiments, gene overexpression or transient transfection of site-mutated plasmids, Western blotting, and mass spectrometry analysis.	Specific acetylation or β-hydroxybutyrylation sites of STAT3

### 6.3 Biomarkers and non-invasive diagnosis

Liver biopsy remains an important reference method for diagnosing MASH and evaluating the degree of liver fibrosis. However, due to its invasive nature, potential complications, and sampling variability, liver biopsy is not suitable for large-scale screening or repeated longitudinal monitoring. Therefore, the development of non-invasive diagnostic approaches is essential for improving the efficiency of MASLD screening, risk stratification, and disease surveillance. Alterations in lysine acylation-related metabolites and acylated proteins may provide new opportunities for the identification of non-invasive biomarkers in MASLD (12, 171).

Serum metabolites represent one class of potential biomarkers. During MASLD progression, metabolic alterations in hepatocytes, macrophages, and hepatic stellate cells can lead to increased intracellular levels of metabolites such as succinate, lactate, and β-hydroxybutyrate, which may subsequently enter the circulation (28, 166, 172). Targeted metabolomics approaches to quantify these circulating metabolites and assess changes in metabolite ratios, such as the lactate/succinate ratio, may indirectly reflect alterations in hepatic metabolic status. Several studies have suggested that these metabolites are associated with hepatic steatosis, insulin resistance, and other metabolic abnormalities (173). However, because individual metabolites generally lack sufficient specificity, their diagnostic performance may require integration with clinical parameters, imaging modalities, and additional biomarker panels.

Another potential class of biomarkers is extracellular vesicles (EVs). Lipotoxicity and inflammatory stimuli can promote the release of EVs from hepatocytes and immune cells (153). EVs contain proteins, lipids, nucleic acids, and metabolism-associated molecules, and their membrane structures can partially protect their cargo from degradation in the circulation. Alterations in glycolytic enzymes, mitochondrial proteins, and their lysine acylation profiles within liver-derived EVs may reflect the metabolic and inflammatory states of hepatic cells (31, 154, 155). By isolating circulating EVs and combining proteomic analysis with protein post-translational modification profiling, it may be possible to characterize EV-associated acylated protein signatures associated with MASLD progression. Integrative analysis of circulating metabolites, EV-derived acylated proteins, and clinical parameters may further facilitate the evaluation of their potential for identifying MASH and predicting liver fibrosis. However, whether these approaches can be effectively applied for MASLD classification, disease progression prediction, and therapeutic response monitoring requires validation in diverse patient populations.

Beyond their mechanistic roles in MASLD progression, lysine acylation-associated molecules may provide clinically relevant biomarkers for disease stratification and therapeutic monitoring (174). Circulating metabolites linked to acyl-CoA metabolism, extracellular vesicle-associated acylated proteins, and liver-derived metabolic signatures may complement existing non-invasive approaches by reflecting metabolic and inflammatory states at the molecular level. However, the clinical application of individual acylation-related biomarkers remains limited by insufficient specificity and the dynamic nature of metabolic modifications (175, 176). Future studies integrating acylome profiling with clinical phenotyping, imaging-based fibrosis assessment, and longitudinal cohorts will be required to establish their prognostic value (177).

### 6.4 Candidate therapeutic targets and delivery strategies

The potential application of lysine acylation-related mechanisms for MASLD intervention depends on the functional evidence supporting relevant targets, the selectivity of therapeutic agents, and the efficiency of delivery strategies. Current studies suggest that acylation regulatory factors, such as SIRT5 and p300/CBP, as well as molecules involved in lactate production and transport, may represent potential targets for further investigation.

Within mitochondria, increased succinylation and malonylation may affect fatty acid oxidation, the tricarboxylic acid cycle, and other metabolic processes. As SIRT5 possesses both desuccinylase and demalonylase activities, modulation of SIRT5 activity may influence the acylation status of proteins such as CPT1A, LCAD, and SDHA, thereby improving mitochondrial metabolic dysfunction (73, 76, 116). However, the target selectivity, pharmacokinetic properties, and long-term safety of SIRT5 modulators require further evaluation.

In addition to mitochondrial targets, nuclear p300/CBP also participates in the regulation of histone acetylation and other lysine acylation modifications. p300/CBP inhibitors, such as A-485, can suppress its catalytic activity and may influence the expression of inflammation- and fibrosis-associated genes in macrophages and hepatic stellate cells (178). Previous studies have suggested that inhibition of p300/CBP may not only affect histone acetylation but also regulate transcriptional programs associated with lactylation (47, 49). However, because p300/CBP participates in multiple physiological processes, systemic inhibition may cause effects in non-target tissues. Therefore, the appropriate dosage, treatment duration, and safety profile of p300/CBP inhibition in MASLD require further investigation.

To enhance hepatic drug distribution and reduce systemic exposure, liver-targeted delivery strategies can be employed. The N-acetylgalactosamine (GalNAc) conjugation technology primarily facilitates the delivery of oligonucleotide therapeutics into hepatocytes by recognizing the asialoglycoprotein receptor (ASGPR) expressed on the hepatocyte surface (179). Therefore, for hepatocyte-related targets such as *Ldha*, GalNAc-conjugated small interfering RNAs (siRNAs) or antisense oligonucleotides may represent potential delivery approaches. However, GalNAc-based delivery mainly targets hepatocytes and has limited efficiency in Kupffer cells, hepatic stellate cells, and liver sinusoidal endothelial cells. Thus, for small-molecule agents such as p300/CBP inhibitors, more suitable liver- or cell-type-specific delivery systems remain to be developed.

Beyond improving hepatic delivery efficiency, future studies should further enhance both site specificity and cell-type selectivity of therapeutic interventions. For example, site-directed mutagenesis, targeted protein degradation technologies, or allosteric regulation approaches may be applied to clarify the functional contribution of specific acylation sites during disease progression. In addition, delivery platforms such as lipid nanoparticles could enable short-term or intermittent intervention at different disease stages. Whether these strategies can minimize effects on non-target hepatic cell populations requires validation in animal models and preclinical studies.

Furthermore, modulation of the gut–liver axis may also influence hepatic acylation-related metabolic processes. Dietary regulation of microbiota-accessible carbohydrates, such as highly esterified pectin and long-chain inulin, can alter gut microbial fermentation products, short-chain fatty acid production, and bile acid metabolism, while improving intestinal barrier integrity and reducing endotoxin burden (31, 173). Engineered bacteria and specific microbial interventions represent promising approaches for regulating intestinal barrier function and gut-derived metabolites; however, their direct effects on hepatic acyltransferases, deacylases and lysine acylation profiles remain insufficiently understood. Therefore, future studies should integrate nutritional intervention, microbiota modulation, and liver-targeted therapeutic delivery strategies to further evaluate their effects on metabolic dysfunction, inflammatory responses, and fibrosis progression in MASLD. Candidate therapeutic targets, delivery strategies, and their current evidence levels are summarized in **Table 5**.

**Table 5:** Candidate therapeutic targets associated with lysine acylation, delivery strategies, and evidence levels in MASLD.

Intervention target	Candidate intervention approaches	Delivery or implementation strategies	Main target sites or cell types	Potential mechanisms of action	Expected research effects	Current evidence basis	Evidence level
SIRT5	Small-molecule SIRT5 activators	Liver-targeted nanodelivery systems or other targeted delivery platforms	Lipotoxicity-induced injured hepatocytes	Modulation of succinylation and malonylation levels of proteins such as CPT1A, LCAD, and SDHA	May improve fatty acid β-oxidation, tricarboxylic acid cycle activity, and mitochondrial metabolism, thereby reducing hepatic lipid accumulation	Mainly supported by mechanistic and preclinical studies; lacking *in vivo* validation of specific acylation sites and confirmation in patient tissues	Level III
p300/CBP	p300/CBP inhibitors such as A-485	Systemic administration or liver-targeted delivery strategies; specific delivery systems require further validation	Hepatocytes; cell-specific delivery strategies are required if targeting HSCs or macrophages	Inhibition of p300/CBP catalytic activity, regulation of histone acetylation and certain lysine acylation modifications, and modulation of inflammation- and fibrosis-related gene expression	May reduce inflammatory responses and expression of pro-fibrotic genes	Mainly supported by cellular and animal studies; direct functional evidence for MASLD-specific acylation sites and cell-type selectivity remains limited	Level III
LDHA	Small-molecule LDHA inhibitors, siRNA, or antisense oligonucleotides targeting Ldha	GalNAc-conjugated oligonucleotides mainly enable hepatocyte delivery; alternative delivery approaches are required for other cell types	Hepatocytes; delivery efficiency in HSCs and macrophages requires independent validation	Reduction of lactate production, potentially decreasing lactyl-CoA formation and histone lysine lactylation levels	May influence lactate accumulation, inflammation-related gene expression, and pro-fibrotic responses	Mainly at the preclinical research stage; *in vivo* functional validation of specific lactylation sites is still lacking	Level III
Gut microbiota and short-chain fatty acid metabolism	Microbiota-accessible carbohydrate formulations, such as highly esterified pectin and long-chain inulin	Oral nutritional intervention or colon-targeted release formulations	Distal colonic microbiota and intestinal barrier	Modulation of short-chain fatty acid and bile acid metabolism, improvement of intestinal barrier function, and reduction of potential endotoxin entry into the portal circulation	May attenuate gut-derived LPS-associated inflammatory responses and indirectly influence hepatic metabolism and protein acylation	Nutritional and microbiota intervention studies provide preliminary support; however, their direct effects on lysine acylation and key acylation targets remain insufficiently validated	Level IV

### 6.5 Unresolved questions and future research priorities

Although increasing evidence indicates that lysine acylation participates in metabolic remodeling, inflammatory activation, and fibrosis progression in MASLD, several important questions remain unresolved. First, the cell-type specificity and temporal dynamics of lysine acylation require further clarification. The liver contains diverse cellular populations, including hepatocytes, Kupffer cells, infiltrating monocyte-derived macrophages, hepatic stellate cells, endothelial cells, and lymphocyte subsets, which experience distinct metabolic environments and inflammatory stimuli (8, 180). Whether the same acylation modification exerts conserved or cell-specific functions in different hepatic cell populations remains largely unknown. Future studies integrating single-cell transcriptomics, spatial multi-omics, and cell-type-resolved proteomic approaches may help define the cellular landscape of acylation remodeling during MASLD progression (164, 181).

Second, the regulatory hierarchy and interaction among different lysine acylation modifications require further investigation. Acetylation, succinylation, malonylation, lactylation, and β-hydroxybutyrylation are closely linked to intracellular metabolic states and share common lysine residues or regulatory enzymes (12, 182). However, whether these modifications compete for specific residues, cooperate to regulate protein function, or represent sequential metabolic responses remains unclear. In addition, the interplay between lysine acylation and other post-translational modifications, including phosphorylation, ubiquitination, and methylation, may represent an important mechanism underlying the complexity of metabolic and inflammatory signaling networks in MASLD.

Third, technical challenges associated with acylation detection need to be carefully considered. Many newly identified acylation events, particularly β-hydroxybutyrylation and lactylation, are currently derived from global proteomic analyses or non-hepatic disease models (58). Although Kbhb has been proposed as a metabolic stress-responsive modification associated with ketone metabolism and transcriptional regulation, its functional interpretation requires caution (28). The detection of Kbhb is technically challenging due to the limited availability and specificity of modification-recognition antibodies, potential cross-reactivity with structurally related acyl modifications, and differences in enrichment strategies among mass spectrometry-based approaches (26, 121). Moreover, changes in cellular β-hydroxybutyrate concentrations do not necessarily indicate site-specific modification changes or establish a causal relationship between Kbhb and downstream biological effects (121). Therefore, findings linking Kbhb to hepatocyte stress responses, senescence-associated secretory phenotype regulation, or transcription factor activity should be validated using orthogonal approaches, including site-specific targeted mass spectrometry, isotope tracing of β-hydroxybutyrate-derived carbon flux, and genetic manipulation of candidate lysine residues (183). Such validation is essential before assigning functional significance to specific Kbhb events in MASLD.

Finally, the clinical relevance of lysine acylation in human MASLD remains to be established. Most current evidence originates from cellular models or experimental animals, whereas longitudinal studies linking specific acylation patterns with disease progression, fibrosis severity, treatment response, or clinical outcomes remain limited (184). Future research should integrate human liver samples, circulating biomarkers, and multi-omics datasets to determine whether acylation signatures can contribute to patient stratification and precision medicine. Collectively, addressing these challenges will facilitate the transition of MASLD-related lysine acylation research from descriptive modification profiling toward mechanistic understanding and clinical translation.

## 7 Conclusion

MASLD progression is influenced by hepatic metabolic dysregulation, immune activation, and alterations in metabolite-driven signaling networks. Lysine acylation represents an important regulatory mechanism linking metabolic states with hepatocyte dysfunction, macrophage activation, and fibrotic responses. Among current findings, acetylation, succinylation, and malonylation of metabolic enzymes, as well as lactylation-related inflammatory regulation, have relatively direct evidence from MASLD models or human liver studies. In contrast, the functional roles of many newly identified acylation events, including β-hydroxybutyrylation and several site-specific modifications, remain largely inferred from other disease contexts and require further validation. Future studies integrating human samples, multi-omics approaches, and site-specific functional experiments are needed to clarify the causal roles of key acylation events and evaluate their potential as biomarkers and therapeutic targets in MASLD.

## Glossary

MASLD: Metabolic dysfunction-associated steatotic liver disease
MASH: Metabolic dysfunction-associated steatohepatitis
NAFLD: Non-alcoholic fatty liver disease
HSCs: Hepatic stellate cells
qHSCs: Quiescent hepatic stellate cells
aHSCs: Activated hepatic stellate cells
LSECs: Liver sinusoidal endothelial cells
Kac: Lysine acetylation
Kla: Lysine lactylation
Ksucc: Lysine succinylation
Kmal: Lysine malonylation
Kbhb: Lysine β-hydroxybutyrylation
Kcr: Lysine crotonylation
PTMs: Post-translational modifications
Acyl-CoA: Acyl-coenzyme A
acetyl-CoA: Acetyl-coenzyme A
succinyl-CoA: Succinyl-coenzyme A
malonyl-CoA: Malonyl-coenzyme A
lactyl-CoA: Lactyl-coenzyme A
βOHB-CoA: β-Hydroxybutyryl-coenzyme A
KATs: Lysine acetyltransferases
HDACs: Histone deacetylases
SIRTs: Sirtuins
SIRT1: Sirtuin 1
SIRT2: Sirtuin 2
SIRT3: Sirtuin 3
SIRT5: Sirtuin 5
SIRT7: Sirtuin 7
p300/CBP: CREB-binding protein/p300 acetyltransferase
HATs: Histone acetyltransferases
DNL: *De novo* lipogenesis
FAO: Fatty acid oxidation
CPT1A: Carnitine palmitoyltransferase 1A
LCAD: Long-chain acyl-CoA dehydrogenase
SDHA: Succinate dehydrogenase flavoprotein subunit A
PDH: Pyruvate dehydrogenase complex
PDHA1: Pyruvate dehydrogenase E1 subunit alpha 1
CPS1: Carbamoyl phosphate synthetase 1
TCA cycle: Tricarboxylic acid cycle
OXPHOS: Oxidative phosphorylation
NAD+: Nicotinamide adenine dinucleotide
ATP: Adenosine triphosphate
ROS: Reactive oxygen species
ER stress: Endoplasmic reticulum stress
UPR: Unfolded protein response
SASP: Senescence-associated secretory phenotype
NF-κB: Nuclear factor kappa-B
RelA/p65: REL-associated protein/p65 subunit of NF-κB
STAT3: Signal transducer and activator of transcription 3
HIF-1α: Hypoxia-inducible factor 1 alpha
NLRP3: NOD-like receptor family pyrin domain-containing 3
ASC: Apoptosis-associated speck-like protein containing a CARD
GSDMD: Gasdermin D
IL-1β: Interleukin-1 beta
IL-6: Interleukin-6
TNF-α: Tumor necrosis factor alpha
TGF-β: Transforming growth factor beta
SMAD2/3: SMAD family member 2/3
α-SMA: Alpha-smooth muscle actin
ECM: Extracellular matrix
LAMs: Lipid-associated macrophages
LPS: Lipopolysaccharide
SUCNR1: Succinate receptor 1
EVs: Extracellular vesicles
MCT1: Monocarboxylate transporter 1
HK2: Hexokinase 2
ALDOA: Aldolase A
m6A: N6-methyladenosine
IGF2BP2: Insulin-like growth factor 2 mRNA-binding protein 2
ICAM-1: Intercellular adhesion molecule-1
VCAM-1: Vascular cell adhesion molecule-1
CCL2: C-C motif chemokine ligand 2
KLF2: Kruppel-like factor 2
eNOS: Endothelial nitric oxide synthase
NO: Nitric oxide
PFKFB3: 6-Phosphofructo-2-kinase/fructose-2,6-bisphosphatase 3
mPTP: Mitochondrial permeability transition pore
GPX4: Glutathione peroxidase 4
ACSL4: Acyl-CoA synthetase long-chain family member 4
CMA: Chaperone-mediated autophagy
GalNAc: N-acetylgalactosamine
ASGPR: Asialoglycoprotein receptor
siRNA: Small interfering RNA
LNPs: Lipid nanoparticles
CRISPR/Cas9: Clustered regularly interspaced short palindromic repeats-associated protein 9
LC-MS/MS: Liquid chromatography-tandem mass spectrometry
EVs: Extracellular vesicles
LC-MS: Liquid chromatography-mass spectrometry
SCFAs: Short-chain fatty acids
MACs: Microbiota-accessible carbohydrates
FXR: Farnesoid X receptor
TGR5: Takeda G protein-coupled bile acid receptor 1
LNP: Lipid nanoparticle
K-to-R: Lysine-to-arginine substitution.

## References

[1] Rinella ME Lazarus JV Ratziu V Francque SM Sanyal AJ Kanwal F . A multisociety delphi consensus statement on new fatty liver disease nomenclature. Hepatology. (2023) 78:1966. doi: 10.1097/HEP.0000000000000520 37363821 PMC10653297

[2] Lee YA Friedman SL . Inflammatory and fibrotic mechanisms in NAFLD—implications for new treatment strategies. J Intern Med. (2022) 291:11–31. doi: 10.1111/joim.13380 34564899 PMC8688191

[3] Loomba R Friedman SL Shulman GI . Mechanisms and disease consequences of nonalcoholic fatty liver disease. Cell. (2021) 184:2537–64. doi: 10.1016/j.cell.2021.04.015 33989548 PMC12168897

[4] Friedman SL Neuschwander-Tetri BA Rinella M Sanyal AJ . Mechanisms of NAFLD development and therapeutic strategies. Nat Med. (2018) 24:908–22. doi: 10.1038/s41591-018-0104-9 29967350 PMC6553468

[5] Neuschwander-Tetri BA . Non-alcoholic fatty liver disease. BMC Med. (2017) 15:45. doi: 10.1186/s12916-017-0806-8 28241825 PMC5330146

[6] Powell EE Wong V-S Rinella M . Non-alcoholic fatty liver disease. Lancet. (2021) 397:2212–24. doi: 10.1016/S0140-6736(20)32511-3 33894145

[7] Schuster S Cabrera D Arrese M Feldstein AE . Triggering and resolution of inflammation in NASH. Nat Rev Gastroenterol Hepatol. (2018) 15:349–64. doi: 10.1038/s41575-018-0009-6 29740166

[8] Krenkel O Tacke F . Liver macrophages in tissue homeostasis and disease. Nat Rev Immunol. (2017) 17:306–21. doi: 10.1038/nri.2017.11 28317925

[9] Tarantino G Citro V Balsano C . Liver-spleen axis in nonalcoholic fatty liver disease. Expert Rev Gastroenterol Hepatol. (2021) 15:759–69. doi: 10.1080/17474124.2021.1914587 33878988

[10] Petta S Gastaldelli A Rebelos E Bugianesi E Messa P Miele L . Pathophysiology of non alcoholic fatty liver disease. Int J Mol Sci. (2016) 17:2082. doi: 10.3390/ijms17122082 27973438 PMC5187882

[11] Zhao S Xu W Jiang W Yu W Lin Y Zhang T . Regulation of cellular metabolism by protein lysine acetylation. Science. (2010) 327:1000–4. doi: 10.1126/science.1179689 20167786 PMC3232675

[12] Sabari BR Zhang D Allis CD Zhao Y . Metabolic regulation of gene expression through histone acylations. Nat Rev Mol Cell Biol. (2017) 18:90–101. doi: 10.1038/nrm.2016.140 27924077 PMC5320945

[13] Barrea L Di Somma C Muscogiuri G Tarantino G Tenore GC Orio F . Nutrition, inflammation and liver-spleen axis. Crit Rev Food Sci Nutr. (2018) 58:3141–58. doi: 10.1080/10408398.2017.1353479 28799803

[14] Heidt T Sager HB Courties G Dutta P Iwamoto Y Zaltsman A . Chronic variable stress activates hematopoietic stem cells. Nat Med. (2014) 20:754–8. doi: 10.1038/nm.3589 24952646 PMC4087061

[15] O’Sullivan D Sanin DE Pearce EJ Pearce EL . Metabolic interventions in the immune response to cancer. Nat Rev Immunol. (2019) 19:324–35. doi: 10.1038/s41577-019-0140-9 30820043

[16] Sreedhar A Wiese EK Hitosugi T . Enzymatic and metabolic regulation of lysine succinylation. Genes Dis. (2020) 7:166–71. doi: 10.1016/j.gendis.2019.09.011 32215286 PMC7083736

[17] Zhao G Zhen J Liu X Guo J Li D Xie J . Protein post-translational modification by lysine succinylation: biochemistry, biological implications, and therapeutic opportunities. Genes Dis. (2023) 10:1242–62. doi: 10.1016/j.gendis.2022.03.009 37397549 PMC10310984

[18] Ghesquière B Wong BW Kuchnio A Carmeliet P . Metabolism of stromal and immune cells in health and disease. Nature. (2014) 511:167–76. doi: 10.1038/nature13312 25008522

[19] Shvedunova M Akhtar A . Modulation of cellular processes by histone and non-histone protein acetylation. Nat Rev Mol Cell Biol. (2022) 23:329–49. doi: 10.1038/s41580-021-00441-y 35042977

[20] Jo C Park S Oh S Choi J Kim E-K Youn H-D . Histone acylation marks respond to metabolic perturbations and enable cellular adaptation. Exp Mol Med. (2020) 52:2005–19. doi: 10.1038/s12276-020-00539-x 33311704 PMC8080766

[21] Bao C Ma Q Ying X Wang F Hou Y Wang D . Histone lactylation in macrophage biology and disease: from plasticity regulation to therapeutic implications. eBioMedicine. (2025) 111:105502. doi: 10.1016/j.ebiom.2024.105502 39662177 PMC11697715

[22] Li Y Feng L Shen Q Jiang X Liu F Zhang C . From metabolic reprogramming to epigenetic modification: association network and targeted treatment strategy between histone lactylation and tumor progression. Front Immunol. (2025) 16. doi: 10.3389/fimmu.2025.1542664 41041328 PMC12484167

[23] Zhao D Guan H Zhao S Mi W Wen H Li Y . YEATS2 is a selective histone crotonylation reader. Cell Res. (2016) 26:629–32. doi: 10.1038/cr.2016.49 27103431 PMC4856769

[24] Li Y Sabari BR Panchenko T Wen H Zhao D Guan H . Molecular coupling of histone crotonylation and active transcription by AF9 YEATS domain. Mol Cell. (2016) 62:181–93. doi: 10.1016/j.molcel.2016.03.028 27105114 PMC4841940

[25] Ren X Zhou Y Xue Z Hao N Li Y Guo X . Histone benzoylation serves as an epigenetic mark for DPF and YEATS family proteins. Nucleic Acids Res. (2021) 49:114–26. doi: 10.1093/nar/gkaa1130 33290558 PMC7797077

[26] Choudhary C Weinert BT Nishida Y Verdin E Mann M . The growing landscape of lysine acetylation links metabolism and cell signalling. Nat Rev Mol Cell Biol. (2014) 15:536–50. doi: 10.1038/nrm3841 25053359

[27] Rho H Terry AR Chronis C Hay N . Hexokinase 2-mediated gene expression via histone lactylation is required for hepatic stellate cell activation and liver fibrosis. Cell Metab. (2023) 35:1406–1423.e8. doi: 10.1016/j.cmet.2023.06.013 37463576 PMC11748916

[28] Zhang D Xie Z Chung D Tang Z Huang H Dai L . Metabolic regulation of gene expression by histone lysine β-hydroxybutyrylation. FASEB J. (2017) 31:755.2–2. doi: 10.1096/fasebj.31.1_supplement.755.2

[29] Peng K Xiao S Xia S Li C Yu H Yu Q . Butyrate inhibits the HDAC8/NF-κB pathway to enhance Slc26a3 expression and improve the intestinal epithelial barrier to relieve colitis. J Agric Food Chem. (2024) 72:24400–16. doi: 10.1021/acs.jafc.4c04456 39440960

[30] Amadieu C Ahmed H Leclercq S Koistinen V Leyrolle Q Stärkel P . Effect of inulin supplementation on fecal and blood metabolome in alcohol use disorder patients: a randomised, controlled dietary intervention. Clin Nutr ESPEN. (2025) 66:361–71. doi: 10.1016/j.clnesp.2025.01.046 39864520

[31] Kwak M-J Park B Choi H Hong W Mun D Son S-H . Gut microbial extracellular vesicles modulate the development of metabolic dysfunction-associated steatohepatitis through the gut-liver axis. Pharmacol Res. (2026) 227:108184. doi: 10.1016/j.phrs.2026.108184 41935802

[32] Hu M Xu Y Zhou H He X . Gut microbial metabolites of amino acids in liver diseases. Gut Microbes. (2025) 17:2586328. doi: 10.1080/19490976.2025.2586328 41277458 PMC12645865

[33] Lin J Nie Q Cheng J Zhong Y-N Zhang T Zhang X . A microbial amino-acid-conjugated bile acid, tryptophan-cholic acid, improves glucose homeostasis via the orphan receptor MRGPRE. Cell. (2025) 188:4530–4548.e25. doi: 10.1016/j.cell.2025.05.010 40446798

[34] Wise JL Cummings BP . The 7-α-dehydroxylation pathway: an integral component of gut bacterial bile acid metabolism and potential therapeutic target. Front Microbiol. (2023) 13. doi: 10.3389/fmicb.2022.1093420 36699589 PMC9868651

[35] García-Ruiz C Baulies A Mari M García-Rovés PM Fernandez-Checa JC . Mitochondrial dysfunction in non-alcoholic fatty liver disease and insulin resistance: cause or consequence? Free Radical Res. (2013) 47:854–68. doi: 10.3109/10715762.2013.830717 23915028

[36] Shum M Ngo J Shirihai OS Liesa M . Mitochondrial oxidative function in NAFLD: friend or foe? Mol Metab. (2021) 50:101134. doi: 10.1016/j.molmet.2020.101134 33276146 PMC8324685

[37] Begriche K Massart J Robin M-A Bonnet F Fromenty B . Mitochondrial adaptations and dysfunctions in nonalcoholic fatty liver disease. Hepatology. (2013) 58:1497–507. doi: 10.1002/hep.26226 23299992

[38] Gao R Li Y Xu Z Zhang F Xu J Hu Y . Mitochondrial pyruvate carrier 1 regulates fatty acid synthase lactylation and mediates treatment of nonalcoholic fatty liver disease. Hepatology. (2023) 78:1800. doi: 10.1097/HEP.0000000000000279 36651176

[39] Habibi M Ferguson D Eichler SJ Chan MM Fu C Pietka TA . A critical role for the mitochondrial pyruvate carrier in hepatic stellate cell activation. Cell Mol Gastroenterol Hepatol. (2025) 19:101517. doi: 10.1016/j.jcmgh.2025.101517 40239806 PMC12166444

[40] Cao H Cai Q Guo W Su Q Qin H Wang T . Malonylation of acetyl-CoA carboxylase 1 promotes hepatic steatosis and is attenuated by ketogenic diet in NAFLD. Cell Rep. (2023) 42:112319. doi: 10.1016/j.celrep.2023.112319 37002924

[41] Guo Y Li J Zhang K . Crotonylation modification and its role in diseases. Front Mol Biosci. (2024) 11. doi: 10.3389/fmolb.2024.1492212 39606030 PMC11599741

[42] Knaap JA Verrijzer CP . Undercover: gene control by metabolites and metabolic enzymes. Genes Dev. (2016) 30:2345–69. doi: 10.1101/gad.289140.116 27881599 PMC5131776

[43] Li X-Y Ji P-X Ni X-X Chen Y-X Sheng L Lian M . Regulation of PPAR-γ activity in lipid-laden hepatocytes affects macrophage polarization and inflammation in nonalcoholic fatty liver disease. World J Hepatol. (2022) 14:1365–81. doi: 10.4254/wjh.v14.i7.1365 36158922 PMC9376780

[44] Goedeke L Bates J Vatner DF Perry RJ Wang T Ramirez R . Acetyl-CoA carboxylase inhibition reverses NAFLD and hepatic insulin resistance but promotes hypertriglyceridemia in rodents. Hepatology. (2018) 68:2197. doi: 10.1002/hep.30097 29790582 PMC6251774

[45] Huang H Zhang D Weng Y Delaney K Tang Z Yan C . The regulatory enzymes and protein substrates for the lysine β-hydroxybutyrylation pathway. Sci Adv. (2021) 7:eabe2771. doi: 10.1126/sciadv.abe2771 33627428 PMC7904266

[46] He M Han Z Liu L Zheng YG . Chemical biology approaches for investigating the functions of lysine acetyltransferases. Angew Chem Int Ed. (2018) 57:1162–84. doi: 10.1002/anie.201704745 28786225

[47] Sabari BR Tang Z Huang H Yong-Gonzalez V Molina H Kong HE . Intracellular crotonyl-CoA stimulates transcription through p300-catalyzed histone crotonylation. Mol Cell. (2015) 58:203–15. doi: 10.1016/j.molcel.2015.02.029 25818647 PMC4501262

[48] Lammers M . Post-translational lysine Ac(et)ylation in bacteria: a biochemical, structural, and synthetic biological perspective. Front Microbiol. (2021) 12. doi: 10.3389/fmicb.2021.757179 34721364 PMC8556138

[49] Luo M . Chemical and biochemical perspectives of protein lysine methylation. Chem Rev. (2018) 118:6656–705. doi: 10.1021/acs.chemrev.8b00008 29927582 PMC6668730

[50] Wellen KE Hatzivassiliou G Sachdeva UM Bui TV Cross JR Thompson CB . ATP-citrate lyase links cellular metabolism to histone acetylation. Science. (2009) 324:1076–80. doi: 10.1126/science.1164097 19461003 PMC2746744

[51] Wagner GR Payne RM . Widespread and enzyme-independent nϵ-acetylation and nϵ-succinylation of proteins in the chemical conditions of the mitochondrial matrix *♦. J Biol Chem. (2013) 288:29036–45. doi: 10.1074/jbc.M113.486753 23946487 PMC3790002

[52] Hou X Zhu L Xu H Shi J Ji S . Dysregulation of protein succinylation and disease development. Front Mol Biosci. (2024) 11. doi: 10.3389/fmolb.2024.1407505 38882606 PMC11176430

[53] Hou X Chen Y Li X Gu X Dong W Shi J . Protein succinylation: regulating metabolism and beyond. Front Nutr. (2024) 11. doi: 10.3389/fnut.2024.1336057 38379549 PMC10876795

[54] Weinert BT Schölz C Wagner SA Iesmantavicius V Su D Daniel JA . Lysine succinylation is a frequently occurring modification in prokaryotes and eukaryotes and extensively overlaps with acetylation. Cell Rep. (2013) 4:842–51. doi: 10.1016/j.celrep.2013.07.024 23954790

[55] Stefanaki C Paltoglou G Mastorakos G Chrousos GP . Chronic stress and steatosis of muscles, bones, liver, and pancreas: a review. Horm Res Paediatr. (2023) 96:66–73. doi: 10.1159/000522540 35144259

[56] Mansouri A Gattolliat C-H Asselah T . Mitochondrial dysfunction and signaling in chronic liver diseases. Gastroenterology. (2018) 155:629–47. doi: 10.1053/j.gastro.2018.06.083 30012333

[57] Du J Zhou Y Su X Yu JJ Khan S Jiang H . Sirt5 is a NAD-dependent protein lysine demalonylase and desuccinylase. Science. (2011) 334:806–9. doi: 10.1126/science.1207861 22076378 PMC3217313

[58] Zhang D Tang Z Huang H Zhou G Cui C Weng Y . Metabolic regulation of gene expression by histone lactylation. Nature. (2019) 574:575–80. doi: 10.1038/s41586-019-1678-1 31645732 PMC6818755

[59] Hirschey MD Shimazu T Goetzman E Jing E Schwer B Lombard DB . SIRT3 regulates mitochondrial fatty-acid oxidation by reversible enzyme deacetylation. Nature. (2010) 464:121–5. doi: 10.1038/nature08778 20203611 PMC2841477

[60] Verdin E . NAD^+^ in aging, metabolism, and neurodegeneration. Science. (2015) 350:1208–13. doi: 10.1126/science.aac4854 26785480

[61] Wu X Liu C Zhang C Kuai L Hu S Jia N . The role of lactate and lactylation in the dysregulation of immune responses in psoriasis. Clin Rev Allergy Immunol. (2025) 68:28. doi: 10.1007/s12016-025-09037-2 40080284

[62] Zhao L Xu S Qiao S Wang Z Wang S Liu C . Myeloid mas drives pyruvate kinase M2-mediated Spi1 lactylation to fuel inflammatory senescence in MASLD. Signal Transduction Targeted Ther. (2026) 11:186. doi: 10.1038/s41392-026-02704-6 42151117 PMC13184135

[63] Tarantino G Citro V . Liver-spleen axis: deciphering a crucial crosstalk in non-alcoholic fatty liver disease progression and therapeutic implications. World J Gastroenterol. (2026) 32(34):119467. doi: 10.3748/wjg.119467

[64] Tarantino G Citro V . Crosstalk between the spleen and other organs/systems: downstream signaling events. Immuno. (2024) 4:479–501. doi: 10.3390/immuno4040030 30654563

[65] Tarantino G Citro V Conforti P Balsano C Capone D . Is there a link between basal metabolic rate, spleen volume and hepatic growth factor levels in patients with obesity-related NAFLD? J Clin Med. (2019) 8:1510. doi: 10.3390/jcm8101510 31547124 PMC6832562

[66] McKim DB Yin W Wang Y Cole SW Godbout JP Sheridan JF . Social stress mobilizes hematopoietic stem cells to establish persistent splenic myelopoiesis. Cell Rep. (2018) 25:2552–2562.e3. doi: 10.1016/j.celrep.2018.10.102 30485819 PMC6342493

[67] Musso G Cassader M Paschetta E Gambino R . Bioactive lipid species and metabolic pathways in progression and resolution of nonalcoholic steatohepatitis. Gastroenterology. (2018) 155:282–302.e8. doi: 10.1053/j.gastro.2018.06.031 29906416

[68] Tarantino G Citro V . What are the common downstream molecular events between alcoholic and nonalcoholic fatty liver? Lipids Health Dis. (2024) 23:41. doi: 10.1186/s12944-024-02031-1 38331795 PMC10851522

[69] Aravinthan A Scarpini C Tachtatzis P Verma S Penrhyn-Lowe S Harvey R . Hepatocyte senescence predicts progression in non-alcohol-related fatty liver disease. J Hepatol. (2013) 58:549–56. doi: 10.1016/j.jhep.2012.10.031 23142622

[70] Papatheodoridi A-M Chrysavgis L Koutsilieris M Chatzigeorgiou A . The role of senescence in the development of nonalcoholic fatty liver disease and progression to nonalcoholic steatohepatitis. Hepatology. (2020) 71:363. doi: 10.1002/hep.30834 31230380

[71] Engelmann C Tacke F . The potential role of cellular senescence in non-alcoholic fatty liver disease. Int J Mol Sci. (2022) 23:652. doi: 10.3390/ijms23020652 35054837 PMC8775400

[72] Ogrodnik M Miwa S Tchkonia T Tiniakos D Wilson CL Lahat A . Cellular senescence drives age-dependent hepatic steatosis. Nat Commun. (2017) 8:15691. doi: 10.1038/ncomms15691 28608850 PMC5474745

[73] Sadhukhan S Liu X Ryu D Nelson OD Stupinski JA Li Z . Metabolomics-assisted proteomics identifies succinylation and SIRT5 as important regulators of cardiac function. Proc Natl Acad Sci. (2016) 113:4320–5. doi: 10.1073/pnas.1519858113 27051063 PMC4843474

[74] Kubatzky KF Gao Y Yu D . Post-translational modulation of cell signalling through protein succinylation. Explor Target Anti-tumor Ther. (2023) 4:1260–85. doi: 10.37349/etat.2023.00196 38213532 PMC10776603

[75] Ke Z Shen K Wang L Xu H Pan X Qian Z . Emerging roles of mitochondrial sirtuin SIRT5 in succinylation modification and cancer development. Front Immunol. (2025) 16. doi: 10.3389/fimmu.2025.1531246 39944690 PMC11814216

[76] Zhao X Yang X Du C Hao H Liu S Liu G . Up-regulated succinylation modifications induce a senescence phenotype in microglia by altering mitochondrial energy metabolism. J Neuroinflamm. (2024) 21:296. doi: 10.1186/s12974-024-03284-4 39543710 PMC11566524

[77] Ferreira-Gonzalez S Lu W-Y Raven A Dwyer B Man TY O’Duibhir E . Paracrine cellular senescence exacerbates biliary injury and impairs regeneration. Nat Commun. (2018) 9:1020. doi: 10.1038/s41467-018-03299-5 29523787 PMC5844882

[78] Wan Y Meng F Wu N Zhou T Venter J Francis H . Substance P increases liver fibrosis by differential changes in senescence of cholangiocytes and hepatic stellate cells. Hepatology. (2017) 66:528–41. doi: 10.1002/hep.29138 28256736 PMC5519428

[79] Zhou T Wu N Meng F Venter J Giang TK Francis H . Knockout of secretin receptor reduces biliary damage and liver fibrosis in Mdr2-/- mice by diminishing senescence of cholangiocytes. Lab Invest. (2018) 98:1449–64. doi: 10.1038/s41374-018-0093-9 29977037 PMC6214714

[80] Cui H Xie N Banerjee S Ge J Jiang D Dey T . Lung myofibroblasts promote macrophage profibrotic activity through lactate-induced histone lactylation. Am J Respir Cell Mol Biol. (2021) 64:115–25. doi: 10.1165/rcmb.2020-0360OC 33074715 PMC7780997

[81] Weinert BT Moustafa T Iesmantavicius V Zechner R Choudhary C . Analysis of acetylation stoichiometry suggests that SIRT3 repairs nonenzymatic acetylation lesions. EMBO J. (2015) 34:2620–32. doi: 10.15252/embj.201591271 26358839 PMC4641529

[82] Hirschey MD Shimazu T Jing E Grueter CA Collins AM Aouizerat B . SIRT3 deficiency and mitochondrial protein hyperacetylation accelerate the development of the metabolic syndrome. Mol Cell. (2011) 44:177–90. doi: 10.1016/j.molcel.2011.07.019 21856199 PMC3563434

[83] Jiang H Khan S Wang Y Charron G He B Sebastian C . SIRT6 regulates TNF-α secretion through hydrolysis of long-chain fatty acyl lysine. Nature. (2013) 496:110–3. doi: 10.1038/nature12038 23552949 PMC3635073

[84] Vaziri H Dessain SK Eaton EN Imai S-I Frye RA Pandita TK . hSIR2SIRT1 functions as an NAD-dependent p53 deacetylase. Cell. (2001) 107:149–59. doi: 10.1016/S0092-8674(01)00527-X 11672523

[85] Wen Y Lambrecht J Ju C Tacke F . Hepatic macrophages in liver homeostasis and diseases-diversity, plasticity and therapeutic opportunities. Cell Mol Immunol. (2021) 18:45–56. doi: 10.1038/s41423-020-00558-8 33041338 PMC7852525

[86] Shan Z Ju C . Hepatic macrophages in liver injury. Front Immunol. (2020) 11. doi: 10.3389/fimmu.2020.00322 32362892 PMC7180226

[87] Liberti MV Locasale JW . Histone lactylation: a new role for glucose metabolism. Trends Biochem Sci. (2020) 45:179–82. doi: 10.1016/j.tibs.2019.12.004 31901298

[88] Xin Q Wang H Li Q Liu S Qu K Liu C . Lactylation: a passing fad or the future of posttranslational modification. Inflammation. (2022) 45:1419–29. doi: 10.1007/s10753-022-01637-w 35224683 PMC9197907

[89] Ye L Jiang Y Zhang M . Crosstalk between glucose metabolism, lactate production and immune response modulation. Cytokine Growth Factor Rev. (2022) 68:81–92. doi: 10.1016/j.cytogfr.2022.11.001 36376165

[90] Yuan H Wu X Wu Q Chatoff A Megill E Gao J . Lysine catabolism reprograms tumour immunity through histone crotonylation. Nature. (2023) 617:818–26. doi: 10.1038/s41586-023-06061-0 37198486 PMC11089809

[91] Hu Y He Z Li Z Wang Y Wu N Sun H . Lactylation: the novel histone modification influence on gene expression, protein function, and disease. Clin Epigenet. (2024) 16:72. doi: 10.1186/s13148-024-01682-2 38812044 PMC11138093

[92] Yu X Yang J Xu J Pan H Wang W Yu X . Histone lactylation: from tumor lactate metabolism to epigenetic regulation. Int J Biol Sci. (2024) 20:1833–54. doi: 10.7150/ijbs.91492 38481814 PMC10929197

[93] Zhou Y Yan J Huang H Liu L Ren L Hu J . The m6A reader IGF2BP2 regulates glycolytic metabolism and mediates histone lactylation to enhance hepatic stellate cell activation and liver fibrosis. Cell Death Dis. (2024) 15:189. doi: 10.1038/s41419-024-06509-9 38443347 PMC10914723

[94] Feng Y Zhang X Wang XJ Chen C . Metabolic reprogramming in fibrosis-related diseases: underlying mechanisms and therapeutics. Mol BioMed. (2026) 7:84. doi: 10.1186/s43556-026-00490-9 42247154 PMC13241376

[95] Min K Yenilmez B Kelly M Echeverria D Elleby M Lifshitz LM . Lactate transporter MCT1 in hepatic stellate cells promotes fibrotic collagen expression in nonalcoholic steatohepatitis. eLife. (2024) 12:RP89136. doi: 10.7554/eLife.89136 38564479 PMC10987092

[96] Gou Y Li A Dong X Hao A Li J Xiang H . Lactate transporter MCT4 regulates the hub genes for lipid metabolism and inflammation to attenuate intracellular lipid accumulation in non-alcoholic fatty liver disease. Genes Dis. (2025) 12:101554. doi: 10.1016/j.gendis.2025.101554 40330148 PMC12052676

[97] Zhang Y Wang H Wang F Guo X Zhao M Zhou Z . Loss of LCAT function aggravates metabolic-associated steatohepatitis (MASH) in golden Syrian hamster. Clin Sci. (2025) 139:1507–25. doi: 10.1042/CS20257764 41195480 PMC12751064

[98] Li Y Kang X Zhou Z Pan L Chen H Liang X . The m6A methyltransferase Mettl3 deficiency attenuates hepatic stellate cell activation and liver fibrosis. Mol Ther. (2022) 30:3714–28. doi: 10.1016/j.ymthe.2022.07.020 35923112 PMC9734030

[99] Li X Li Y Zhang W Jiang F Lin L Wang Y . The IGF2BP3/notch/Jag1 pathway: a key regulator of hepatic stellate cell ferroptosis in liver fibrosis. Clin Transl Med. (2024) 14:e1793. doi: 10.1002/ctm2.1793 39113232 PMC11306284

[100] Liu W-W Zheng S-Q Li T Fei Y-F Wang C Zhang S . RNA modifications in cellular metabolism: implications for metabolism-targeted therapy and immunotherapy. Signal Transduction Targeted Ther. (2024) 9:70. doi: 10.1038/s41392-024-01777-5 38531882 PMC10966055

[101] Liu S Ma Q Zeng C Li H Su J Song Z . Crosstalk between lactylation and RNA modifications in tumorigenesis: mechanisms and therapeutic implications. biomark Res. (2025) 13:110. doi: 10.1186/s40364-025-00824-9 40859309 PMC12382095

[102] Yao Q Chen Y Zhang X Wang L Li J Lv J . Decoding m6A RNA methylation in kidney disorders: from molecular insights to therapeutic strategies. J Transl Med. (2025) 23:771. doi: 10.1186/s12967-025-06817-4 40640897 PMC12247441

[103] Kisseleva T Ganguly S Murad R Wang A Brenner DA . Regulation of hepatic stellate cell phenotypes in metabolic dysfunction–associated steatohepatitis. Gastroenterology. (2025) 169:797–812. doi: 10.1053/j.gastro.2025.03.010 40120772 PMC12416914

[104] Felli E Nulan Y Ortega-Ribera M Fernández-Iglesias A Gracia-Sancho J . The role of liver sinusoidal endothelial cells in liver diseases: key players in health and pathology. J Hepatol. (2026) 84:655–72. doi: 10.1016/j.jhep.2025.10.017 42133683

[105] Marrone G Russo L Rosado E Hide D García-Cardeña G García-Pagán JC . The transcription factor KLF2 mediates hepatic endothelial protection and paracrine endothelial–stellate cell deactivation induced by statins. J Hepatol. (2013) 58:98–103. doi: 10.1016/j.jhep.2012.08.026 22989565

[106] Dai Q Qing X Jiang W Wang S Liu S Liu X . Aging aggravates liver fibrosis through downregulated hepatocyte SIRT1-induced liver sinusoidal endothelial cell dysfunction. Hepatol Commun. (2024) 8:e0350. doi: 10.1097/HC9.0000000000000350 38126919 PMC10749712

[107] DeLeve LD . Liver sinusoidal endothelial cells in hepatic fibrosis. Hepatology. (2015) 61:1740. doi: 10.1002/hep.27376 25131509 PMC4333127

[108] Doddaballapur A Michalik KM Manavski Y Lucas T Houtkooper RH You X . Laminar shear stress inhibits endothelial cell metabolism via KLF2-mediated repression of PFKFB3. Arterioscler Thromb Vasc Biol. (2015) 35:137–45. doi: 10.1161/ATVBAHA.114.304277 25359860

[109] Mattagajasingh I Kim C-S Naqvi A Yamamori T Hoffman TA Jung S-B . SIRT1 promotes endothelium-dependent vascular relaxation by activating endothelial nitric oxide synthase. Proc Natl Acad Sci. (2007) 104:14855–60. doi: 10.1073/pnas.0704329104 17785417 PMC1976244

[110] Ramirez T Li Y-M Yin S Xu M-J Feng D Zhou Z . Aging aggravates alcoholic liver injury and fibrosis in mice by downregulating sirtuin 1 expression. J Hepatol. (2017) 66:601–9. doi: 10.1016/j.jhep.2016.11.004 27871879 PMC5316497

[111] Luo X Bai Y He S Sun S Jiang X Yang Z . Sirtuin 1 ameliorates defenestration in hepatic sinusoidal endothelial cells during liver fibrosis via inhibiting stress-induced premature senescence. Cell Proliferation. (2021) 54:e12991. doi: 10.1111/cpr.12991 33522656 PMC7941223

[112] Di Pascoli M Diví M Rodríguez-Vilarrupla A Rosado E Gracia-Sancho J Vilaseca M . Resveratrol improves intrahepatic endothelial dysfunction and reduces hepatic fibrosis and portal pressure in cirrhotic rats. J Hepatol. (2013) 58:904–10. doi: 10.1016/j.jhep.2012.12.012 23262250

[113] Marrone G Maeso-Díaz R García-Cardena G Abraldes JG García-Pagán JC Bosch J . KLF2 exerts antifibrotic and vasoprotective effects in cirrhotic rat livers: behind the molecular mechanisms of statins. Gut. (2015) 64(9):1434–43. doi: 10.1136/gutjnl-2014-308338 25500203

[114] Zeng H Pan T Zhan M Hailiwu R Liu B Yang H . Suppression of PFKFB3-driven glycolysis restrains endothelial-to-mesenchymal transition and fibrotic response. Signal Transduction Targeted Ther. (2022) 7:303. doi: 10.1038/s41392-022-01097-6 36045132 PMC9433407

[115] Liu X Wei W Liu Y Yang X Wu J Zhang Y . MOF as an evolutionarily conserved histone crotonyltransferase and transcriptional activation by histone acetyltransferase-deficient and crotonyltransferase-competent CBP/p300. Cell Discov. (2017) 3:17016. doi: 10.1038/celldisc.2017.16 28580166 PMC5441097

[116] Rardin MJ He W Nishida Y Newman JC Carrico C Danielson SR . SIRT5 regulates the mitochondrial lysine succinylome and metabolic networks. Cell Metab. (2013) 18:920–33. doi: 10.1016/j.cmet.2013.11.013 24315375 PMC4105152

[117] Zhou Y Zhang H He B Du J Lin H Cerione RA . The bicyclic intermediate structure provides insights into the desuccinylation mechanism of human sirtuin 5 (SIRT5)*. J Biol Chem. (2012) 287:28307–14. doi: 10.1074/jbc.M112.384511 22767592 PMC3436533

[118] Park J Chen Y Tishkoff DX Peng C Tan M Dai L . SIRT5-mediated lysine desuccinylation impacts diverse metabolic pathways. Mol Cell. (2013) 50:919–30. doi: 10.1016/j.molcel.2013.06.001 23806337 PMC3769971

[119] Nishida Y Rardin MJ Carrico C He W Sahu AK Gut P . SIRT5 regulates both cytosolic and mitochondrial protein malonylation with glycolysis as a major target. Mol Cell. (2015) 59:321–32. doi: 10.1016/j.molcel.2015.05.022 26073543 PMC4571487

[120] Zhang Y Bharathi SS Rardin MJ Lu J Maringer KV Sims-Lucas S . Lysine desuccinylase SIRT5 binds to cardiolipin and regulates the electron transport chain. J Biol Chem. (2017) 292:10239–49. doi: 10.1074/jbc.M117.785022 28458255 PMC5473227

[121] Carrico C Meyer JG He W Gibson BW Verdin E . The mitochondrial acylome emerges: proteomics, regulation by sirtuins, and metabolic and disease implications. Cell Metab. (2018) 27:497–512. doi: 10.1016/j.cmet.2018.01.016 29514063 PMC5863732

[122] Finley LWS Haas W Desquiret-Dumas V Wallace DC Procaccio V Gygi SP . Succinate dehydrogenase is a direct target of sirtuin 3 deacetylase activity. PloS One. (2011) 6:e23295. doi: 10.1371/journal.pone.0023295 21858060 PMC3157345

[123] Ozden O Park S-H Wagner BA Yong Song H Zhu Y Vassilopoulos A . SIRT3 deacetylates and increases pyruvate dehydrogenase activity in cancer cells. Free Radical Biol Med. (2014) 76:163–72. doi: 10.1016/j.freeradbiomed.2014.08.001 25152236 PMC4364304

[124] Jing E O’Neill BT Rardin MJ Kleinridders A Ilkeyeva OR Ussar S . Sirt3 regulates metabolic flexibility of skeletal muscle through reversible enzymatic deacetylation. Diabetes. (2013) 62:3404–17. doi: 10.2337/db12-1650 23835326 PMC3781465

[125] Fan J Shan C Kang H-B Elf S Xie J Tucker M . Tyr phosphorylation of PDP1 toggles recruitment between ACAT1 and SIRT3 to regulate the pyruvate dehydrogenase complex. Mol Cell. (2014) 53:534–48. doi: 10.1016/j.molcel.2013.12.026 24486017 PMC3943932

[126] Nakagawa T Lomb DJ Haigis MC Guarente L . SIRT5 deacetylates carbamoyl phosphate synthetase 1 and regulates the urea cycle. Cell. (2009) 137:560–70. doi: 10.1016/j.cell.2009.02.026 19410549 PMC2698666

[127] Gallego-Durán R Ampuero J Pastor-Ramírez H Álvarez-Amor L del Campo JA Maya-Miles D . Liver injury in non-alcoholic fatty liver disease is associated with urea cycle enzyme dysregulation. Sci Rep. (2022) 12:3418. doi: 10.1038/s41598-022-06614-9 35232986 PMC8888708

[128] Luukkonen PK Qadri S Ahlholm N Porthan K Männistö V Sammalkorpi H . Distinct contributions of metabolic dysfunction and genetic risk factors in the pathogenesis of non-alcoholic fatty liver disease. J Hepatol. (2022) 76:526–35. doi: 10.1016/j.jhep.2021.10.013 34710482 PMC8852745

[129] Yeung F Hoberg JE Ramsey CS Keller MD Jones DR Frye RA . Modulation of NF-κB-dependent transcription and cell survival by the SIRT1 deacetylase. EMBO J. (2004) 23:2369–80. doi: 10.1038/sj.emboj.7600244 15152190 PMC423286

[130] Yuan Z Guan Y Chatterjee D Chin YE . Stat3 dimerization regulated by reversible acetylation of a single lysine residue. Science. (2005) 307:269–73. doi: 10.1126/science.1105166 15653507

[131] He M Chiang H-H Luo H Zheng Z Qiao Q Wang L . An acetylation switch of the NLRP3 inflammasome regulates aging-associated chronic inflammation and insulin resistance. Cell Metab. (2020) 31:580–591.e5. doi: 10.1016/j.cmet.2020.01.009 32032542 PMC7104778

[132] Youm Y-H Grant RW McCabe LR Albarado DC Nguyen KY Ravussin A . Canonical Nlrp3 inflammasome links systemic low-grade inflammation to functional decline in aging. Cell Metab. (2013) 18:519–32. doi: 10.1016/j.cmet.2013.09.010 24093676 PMC4017327

[133] Liang R Qi X Cai Q Niu L Huang X Zhang D . The role of NLRP3 inflammasome in aging and age-related diseases. Immun Ageing. (2024) 21:14. doi: 10.1186/s12979-023-00395-z 38317229 PMC10840156

[134] Huang J Wang Y Hu H He K Jiang X Huang R . SIRT5 safeguards against T-2 toxin induced liver injury by repressing iron accumulation, oxidative stress, and the activation of NLRP3 inflammasome. Toxicol Appl Pharmacol. (2024) 492:117084. doi: 10.1016/j.taap.2024.117084 39241930

[135] Wu M Ye X . Quercetin-4′-O-β-D-glucopyranoside inhibits podocyte injury by SIRT5-mediated desuccinylation of NEK7. Available online at: https://onlinelibrary.wiley.com/doi/10.1111/1440-1681.13909 (Accessed June 14, 2026). 10.1111/1440-1681.13909 39038854

[136] Inoue Y Itoh Y Abe K Okamoto T Daitoku H Fukamizu A . Smad3 is acetylated by p300/CBP to regulate its transactivation activity. Oncogene. (2007) 26:500–8. doi: 10.1038/sj.onc.1209826 16862174

[137] Tu AW Luo K . Acetylation of Smad2 by the Co-activator p300 regulates activin and transforming growth factor β response *. J Biol Chem. (2007) 282:21187–96. doi: 10.1074/jbc.M700085200 17478422

[138] Simonsson M Kanduri M Grönroos E Heldin C-H Ericsson J . The DNA binding activities of Smad2 and Smad3 are regulated by coactivator-mediated acetylation *. J Biol Chem. (2006) 281:39870–80. doi: 10.1074/jbc.M607868200 17074756

[139] Itzen F Greifenberg AK Bösken CA Geyer M . Brd4 activates P-TEFb for RNA polymerase II CTD phosphorylation. Nucleic Acids Res. (2014) 42:7577–90. doi: 10.1093/nar/gku449 24860166 PMC4081074

[140] Yang Z Yik JHN Chen R He N Jang MK Ozato K . Recruitment of P-TEFb for stimulation of transcriptional elongation by the bromodomain protein Brd4. Mol Cell. (2005) 19:535–45. doi: 10.1016/j.molcel.2005.06.029 16109377

[141] Schröder S Cho S Zeng L Zhang Q Kaehlcke K Mak L . Two-pronged binding with bromodomain-containing protein 4 liberates positive transcription elongation factor b from inactive ribonucleoprotein complexes *. J Biol Chem. (2012) 287:1090–9. doi: 10.1074/jbc.M111.282855 22084242 PMC3256921

[142] Chen C Wang D Yu Y Zhao T Min N Wu Y . Legumain promotes tubular ferroptosis by facilitating chaperone-mediated autophagy of GPX4 in AKI. Cell Death Dis. (2021) 12:65. doi: 10.1038/s41419-020-03362-4 33431801 PMC7801434

[143] Wu Z Geng Y Lu X Shi Y Wu G Zhang M . Chaperone-mediated autophagy is involved in the execution of ferroptosis. Proc Natl Acad Sci. (2019) 116:2996–3005. doi: 10.1073/pnas.1819728116 30718432 PMC6386716

[144] Yu S Li Z Zhang Q Wang R Zhao Z Ding W . GPX4 degradation via chaperone-mediated autophagy contributes to antimony-triggered neuronal ferroptosis. Ecotoxicol Environ Saf. (2022) 234:113413. doi: 10.1016/j.ecoenv.2022.113413 35305351

[145] Wang T Liu X Feng X Zhang Z Lv R Feng W . GPX4 degradation contributes to heat stress-induced liver injury via chaperone-mediated autophagy. Biochim Biophys Acta (BBA) - Mol Cell Res. (2025) 1872:119988. doi: 10.1016/j.bbamcr.2025.119988 40368268

[146] Zhou P Peng X Zhang K Cheng J Tang M Shen L . HAT1/HDAC2 mediated ACSL4 acetylation confers radiosensitivity by inducing ferroptosis in nasopharyngeal carcinoma. Cell Death Dis. (2025) 16:160. doi: 10.1038/s41419-025-07477-4 40050614 PMC11885570

[147] Lu W Deng Y Liu M Hu Y Yang K Wang B . Regulation of apoptosis, ferroptosis, and pyroptosis mediated by acetylation. Cell Death Discov. (2026) 12:15. doi: 10.1038/s41420-025-02859-1 41513612 PMC12789129

[148] Yu Q Yang D Ran B Pan J Song Y Cui M . SIRT4-mediated deacetylation of PRDX3 attenuates liver ischemia reperfusion injury by suppressing ferroptosis. Int J Biol Sci. (2025) 21:4663–82. doi: 10.7150/ijbs.114510 40765819 PMC12320503

[149] Place DE Kanneganti T-D . Metabolic regulation of pyroptotic cell death expands the therapeutic landscape for treating inflammatory disease. Signal Transduction Targeted Ther. (2021) 6:37. doi: 10.1038/s41392-021-00467-w 33514689 PMC7846556

[150] Kruse J-P Gu W . Modes of p53 regulation. Cell. (2009) 137:609–22. doi: 10.1016/j.cell.2009.04.050 19450511 PMC3737742

[151] Littlewood-Evans A Sarret S Apfel V Loesle P Dawson J Zhang J . GPR91 senses extracellular succinate released from inflammatory macrophages and exacerbates rheumatoid arthritis. J Exp Med. (2016) 213:1655–62. doi: 10.1084/jem.20160061 27481132 PMC4995082

[152] Trauelsen M Hiron TK Lin D Petersen JE Breton B Husted AS . Extracellular succinate hyperpolarizes M2 macrophages through SUCNR1/GPR91-mediated gq signaling. Cell Rep. (2021) 35:109246. doi: 10.1016/j.celrep.2021.109246 34133934

[153] Guo Q Furuta K Lucien F Gutierrez Sanchez LH Hirsova P Krishnan A . Integrin β1-enriched extracellular vesicles mediate monocyte adhesion and promote liver inflammation in murine NASH. J Hepatol. (2019) 71:1193–205. doi: 10.1016/j.jhep.2019.07.019 31433301 PMC6864271

[154] Hirsova P Ibrahim SH Krishnan A Verma VK Bronk SF Werneburg NW . Lipid-induced signaling causes release of inflammatory extracellular vesicles from hepatocytes. Gastroenterology. (2016) 150:956–67. doi: 10.1053/j.gastro.2015.12.037 26764184 PMC4808464

[155] Jiang F Chen Q Wang W Ling Y Yan Y Xia P . Hepatocyte-derived extracellular vesicles promote endothelial inflammation and atherogenesis via microRNA-1. J Hepatol. (2020) 72:156–66. doi: 10.1016/j.jhep.2019.09.014 31568800

[156] Liao C-Y Song MJ Gao Y Mauer AS Revzin A Malhi H . Hepatocyte-derived lipotoxic extracellular vesicle sphingosine 1-phosphate induces macrophage chemotaxis. Front Immunol. (2018) 9. doi: 10.3389/fimmu.2018.02980 30619336 PMC6305739

[157] Povero D Eguchi A Li H Johnson CD PapouChado BG Wree A . Circulating extracellular vesicles with specific proteome and liver MicroRNAs are potential biomarkers for liver injury in experimental fatty liver disease. PloS One. (2014) 9:e113651. doi: 10.1371/journal.pone.0113651 25470250 PMC4254757

[158] Hirsova P Ibrabim SH Gores GJ Malhi H . Lipotoxic lethal and sublethal stress signaling in hepatocytes: relevance to NASH pathogenesis [S. J Lipid Res. (2016) 57:1758–70. doi: 10.1194/jlr.R066357 27049024 PMC5036373

[159] Sunny NE Parks EJ Browning JD Burgess SC . Excessive hepatic mitochondrial TCA cycle and gluconeogenesis in humans with nonalcoholic fatty liver disease. Cell Metab. (2011) 14:804–10. doi: 10.1016/j.cmet.2011.11.004 22152305 PMC3658280

[160] Tacke F . Targeting hepatic macrophages to treat liver diseases. J Hepatol. (2017) 66:1300–12. doi: 10.1016/j.jhep.2017.02.026 28267621

[161] Tannahill GM Curtis AM Adamik J Palsson-McDermott EM McGettrick AF Goel G . Succinate is an inflammatory signal that induces IL-1β through HIF-1α. Nature. (2013) 496:238–42. doi: 10.1038/nature11986 23535595 PMC4031686

[162] Honda M Kubes P . Neutrophils and neutrophil extracellular traps in the liver and gastrointestinal system. Nat Rev Gastroenterol Hepatol. (2018) 15:206–21. doi: 10.1038/nrgastro.2017.183 29382950

[163] Pfister D Núñez NG Pinyol R Govaere O Pinter M Szydlowska M . NASH limits anti-tumour surveillance in immunotherapy-treated HCC. Nature. (2021) 592:450–6. doi: 10.1038/s41586-021-03362-0 33762733 PMC8046670

[164] Guilliams M Bonnardel J Haest B Vanderborght B Wagner C Remmerie A . Spatial proteogenomics reveals distinct and evolutionarily conserved hepatic macrophage niches. Cell. (2022) 185:379–396.e38. doi: 10.1016/j.cell.2021.12.018 35021063 PMC8809252

[165] Tsuchida T Friedman SL . Mechanisms of hepatic stellate cell activation. Nat Rev Gastroenterol Hepatol. (2017) 14:397–411. doi: 10.1038/nrgastro.2017.38 28487545

[166] Buchanan JL Taylor EB . Mitochondrial pyruvate carrier function in health and disease across the lifespan. Biomolecules. (2020) 10:1162. doi: 10.3390/biom10081162 32784379 PMC7464753

[167] Weiss CAM Brown LA Miranda L Pellizzoni P Steigerwald S Remmert K . Single-cell spatial proteomics maps human liver zonation patterns and their vulnerability to disruption in tissue architecture. Nat Metab. (2026) 8:741–56. doi: 10.1038/s42255-026-01459-2 41721135 PMC13031132

[168] Rosenberger FA Thielert M Strauss MT Schweizer L Ammar C Mädler SC . Spatial single-cell mass spectrometry defines zonation of the hepatocyte proteome. Nat Methods. (2023) 20:1530–6. doi: 10.1038/s41592-023-02007-6 37783884 PMC10555842

[169] Rosenberger FA Mädler SC Thorhauge KH Steigerwald S Fromme M Lebedev M . Deep visual proteomics maps proteotoxicity in a genetic liver disease. Nature. (2025) 642:484–91. doi: 10.1038/s41586-025-08885-4 40240610 PMC12158776

[170] Kim Y Hong S-A Yu J Eom J Jang K Yoon S . Adenine base editing and prime editing of chemically derived hepatic progenitors rescue genetic liver disease. Cell Stem Cell. (2021) 28:1614–1624.e5. doi: 10.1016/j.stem.2021.04.010 33951479

[171] Hotamisligil GS . Foundations of immunometabolism and implications for metabolic health and disease. Immunity. (2017) 47:406–20. doi: 10.1016/j.immuni.2017.08.009 28930657 PMC5627521

[172] Tan Q Liu M Tao X . Targeting lactylation: from metabolic reprogramming to precision therapeutics in liver diseases. Biomolecules. (2025) 15:1178. doi: 10.3390/biom15081178 40867622 PMC12385171

[173] Ni Y Qian L Siliceo SL Long X Nychas E Liu Y . Resistant starch decreases intrahepatic triglycerides in patients with NAFLD via gut microbiome alterations. Cell Metab. (2023) 35:1530–1547.e8. doi: 10.1016/j.cmet.2023.08.002 37673036

[174] Wattacheril JJ Abdelmalek MF Lim JK Sanyal AJ . AGA clinical practice update on the role of noninvasive biomarkers in the evaluation and management of nonalcoholic fatty liver disease: expert review. Gastroenterology. (2023) 165:1080–8. doi: 10.1053/j.gastro.2023.06.013 37542503

[175] Li Y Son K Serna-Salas S Wolters JC Wassenaar NPM Driessen S . Plasma extracellular vesicles contain protein biomarkers for capturing stages of metabolic dysfunction-associated steatotic liver disease: a preliminary exploratory study. Biomolecules. (2025) 15:1596. doi: 10.3390/biom15111596 41301514 PMC12650337

[176] Povero D Yamashita H Ren W Subramanian MG Myers RP Eguchi A . Characterization and proteome of circulating extracellular vesicles as potential biomarkers for NASH. Hepatol Commun. (2020) 4:1263–78. doi: 10.1002/hep4.1556 32923831 PMC7471415

[177] Tincopa MA Loomba R . Non-invasive diagnosis and monitoring of non-alcoholic fatty liver disease and non-alcoholic steatohepatitis. Lancet Gastroenterol Hepatol. (2023) 8:660–70. doi: 10.1016/S2468-1253(23)00066-3 37060912

[178] Lasko LM Jakob CG Edalji RP Qiu W Montgomery D Digiammarino EL . Discovery of a selective catalytic p300/CBP inhibitor that targets lineage-specific tumours. Nature. (2017) 550:128–32. doi: 10.1038/nature24028 28953875 PMC6050590

[179] Nair JK Willoughby JLS Chan A Charisse K Alam M Wang Q . Multivalent N-acetylgalactosamine-conjugated siRNA localizes in hepatocytes and elicits robust RNAi-mediated gene silencing. J Am Chem Soc. (2014) 136:16958–61. doi: 10.1021/ja505986a 25434769

[180] Ramachandran P Dobie R Wilson-Kanamori JR Dora EF Henderson BEP Luu NT . Resolving the fibrotic niche of human liver cirrhosis at single-cell level. Nature. (2019) 575:512–8. doi: 10.1038/s41586-019-1631-3 31597160 PMC6876711

[181] Ramachandran P Matchett KP Dobie R Wilson-Kanamori JR Henderson NC . Single-cell technologies in hepatology: new insights into liver biology and disease pathogenesis. Nat Rev Gastroenterol Hepatol. (2020) 17:457–72. doi: 10.1038/s41575-020-0304-x 32483353

[182] Xie Z Zhang D Chung D Tang Z Huang H Dai L . Metabolic regulation of gene expression by histone lysine β-hydroxybutyrylation. Mol Cell. (2016) 62:194–206. doi: 10.1016/j.molcel.2016.03.036 27105115 PMC5540445

[183] Govaere O Cockell S Tiniakos D Queen R Younes R Vacca M . Transcriptomic profiling across the nonalcoholic fatty liver disease spectrum reveals gene signatures for steatohepatitis and fibrosis. Sci Transl Med. (2020) 12:eaba4448. doi: 10.1126/scitranslmed.aba4448 33268509

[184] Anstee QM Reeves HL Kotsiliti E Govaere O Heikenwalder M . From NASH to HCC: current concepts and future challenges. Nat Rev Gastroenterol Hepatol. (2019) 16:411–28. doi: 10.1038/s41575-019-0145-7 31028350

